# Lysosome-dependent cell death in hepatocellular carcinoma: unlocking the therapeutic potential of natural products

**DOI:** 10.3389/fphar.2026.1859886

**Published:** 2026-06-15

**Authors:** Yijing Xu, Ruixin Wang, Xiaofang Xie, Huali Fan, Fu Peng

**Affiliations:** 1 West China School of Pharmacy, Department of Pharmacy, West China Tianfu Hospital, West China Hospital, Sichuan University, Chengdu, China; 2 Chinese Medicine Germplasm Resources Innovation and Effective Uses Key Laboratory of Sichuan Province, State Key Laboratory of Southwestern Chinese Medicine Resources, Chengdu University of Traditional Chinese Medicine, Chengdu, China

**Keywords:** cancer therapy, HCC, lysosomal membrane permeabilization (lmp), lysosome-dependent cell death (LDCD), natural product

## Abstract

Hepatocellular carcinoma (HCC) is one of the deadliest malignant tumors in the world, and the available targeted therapies (e.g., sorafenib, lenvatinib) have limited options and frequent drug resistance. Lysosome-dependent cell death (LDCD), characterized by increased lysosomal membrane permeabilization (LMP) and the release of proteases, has attracted considerable attention as a non-apoptotic mechanism that can circumvent drug resistance. In recent years, researchers have used natural compounds in the treatment of HCC, which effectively induce LDCD through a variety of mechanisms, such as acid sphingomyelinase inhibition, lysosomal-iron-ferroptosis axis activation, lysosomal pH regulation, PI3K/AKT/mTOR-TFEB pathway inhibition and so on. These compounds synergize with conventional targeted agents to overcome drug resistance through direct cytotoxicity or targeting hypertrophic lysosomal drug release. This article reviews the regulation of LDCD and the role of natural products in HCC based on PubMed, Web of Science and CNKI databases, aiming to providing a reference for the treatment of drug-resistant liver cancer.

## 1 Introduction

### 1.1 Hepatocellular carcinoma

Hepatocellular carcinoma (HCC) represents one of the most formidable challenges in oncology, ranking as the third leading cause of cancer-related mortality worldwide and primarily affects individuals with cirrhosis (Mauro et al., 2025). Based on the GLOBOCAN 2022 estimation, liver cancer accounted for 865,269 new cases, ranking sixth and 757,948 deaths globally, with China bearing nearly 42.5% of this burden (Bray et al., 2024). The burden of disease exhibits significant heterogeneity across geographic regions and populations. About 70% of liver cancer cases worldwide occur in East Asia, Southeast Asia and Africa. In contrast, the incidence of liver cancer is lower in the Americas and Europe (Mauro et al., 2025). Besides, the incidence and mortality of HCC in men are 2–4 times higher than in women (Ho et al., 2024).

**FIGURE 1:**
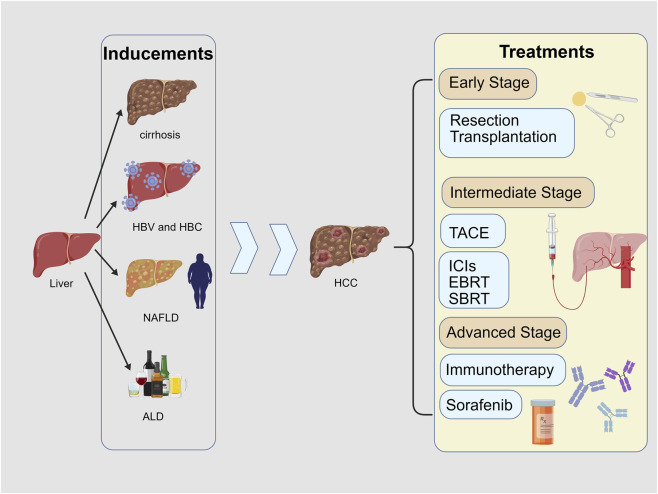
The epidemiology and main therapeutic strategies of HCC. Supported by BioGDP (BioGDP - Generic Diagramming Platform for Biomedical Graphics).

Most HCCs occur against the background of liver disease, whether accompanied by cirrhosis or not (Deshpande et al., 2011). Chronic hepatitis B virus (HBV) and hepatitis C virus (HCV) infection remain the leading causes of HCC worldwide, accounting for about 21%–55% of global HCC cases (Sequeira et al., 2025). With the popularization of vaccination coverage and the application of direct-acting antivirals, the risk of virus-related liver cancer has decreased significantly (Hwang et al., 2025). However, recent investigations show that the incidence of metabolic syndrome and its associated metabolic-associated fatty liver disease (MAFLD) and alcohol-associated liver disease (ALD) is rising (Hwang et al., 2025). This shift is attributable to the increase in obesity, type 2 diabetes, and excessive alcohol consumption. The latest data shows that the number of cases of HCC related to metabolic abnormalities is increasing rapidly, which becomes a fastest-growing etiology worldwide (Wu S. et al., 2025) (Figure 1).

Treatments for HCC adopt a staging and individualized protocol, and strategies are based on a combination of tumor burden, liver function reserve and patient performance status. Surgical resection and liver transplantation are the main curative methods for early HCC, but donor shortages limit liver transplantation applications (Shen et al., 2016). However, organ shortages have limited their widespread use, and ultimately, only about 10% of HCC patients receive transplants. For patients with intermediate-stage HCC, transarterial chemoembolization (TACE) is the standard treatment, and a variety of new local therapies and immunotherapies such as transarterial radioembolization (TARE) with yttrium-90 (90Y) microspheres (Yu Q. et al., 2023), immune checkpoint inhibitors (ICIs) (Li L. et al., 2025), external beam radiation (EBRT) and stereotactic body radiation therapy (SBRT) (Liu et al., 2023) have proven effective in intermediate-stage and not suitable for surgical removal cases. For advanced HCC, therapeutics shift from single-agent multi-kinase inhibitors to combination immunotherapy regimens. Sorafenib, a tyrosine kinase inhibitor, remains the first-line option. However, patients usually develop drug resistance within 6 months by various mechanisms (Courtois et al., 2026), such as aberrant epigenetic regulation and activation of alternative survival pathways (PI3K/AKT, JAK/STAT) (Zhang N. et al., 2025). The combination of atezomab and bevacizumab (AB) is proved more effective (Sonbol et al., 2020) and the combination immunotherapy with anti-PD-L1 and anti-CTLA-4 antibodies was approved as second-line therapy (Kudo, 2022). Despite these advances, the median survival is still less than 2 years, and many patients develop primary or acquired resistance (Joerg et al., 2023). The emergence of drug resistance, particularly through the increase of anti-apoptotic proteins (BCL-2) and decrease of pro-apoptotic proteins (PUMA, BIM), has made apoptosis-dependent therapies increasingly ineffective (Qi et al., 2025). Emerging therapies such as CAR-T (Zhou et al., 2024), ICIs like programmed cell death protein 1 (PD-1) and programmed death receptor-ligand 1 (PD-L1) inhibitors and targeting sodium transporters with specific inhibitors (An et al., 2026) provide new directions for HCC treatments (Liu et al., 2021). However, due to factors such as tumor heterogeneity, liver cirrhosis, immune-related adverse events (Chen et al., 2022) and drug resistance, the overall prognosis of advanced HCC is still poor.

Therefore, there is an urgent need to find innovative treatment modalities to avoid drug resistance, selectively eliminate malignant hepatocytes while preserving normal parenchyma, and improve the outcome of this fatal malignancy.

### 1.2 Lysosomal membrane permeabilization (LMP) and lysosome-dependent cell death (LDCD)

Targeting the pathways of programmed cell death has become a hot topic in anticancer research, with the role of lysosomes in the regulation of cell death receiving widespread attention in recent years. The lysosome, traditionally regarded as the “recycling center” of the cell due to its roles in macromolecular degradation, has emerged as a sophisticated signaling hub that plays a pivotal role in regulating cell fate decisions. Beyond its canonical degradative functions, the lysosome serves as an integration point for metabolic stress, nutrient availability, and cell death signaling (Zhang R. et al., 2025). Central to this paradigm shift is the phenomenon of lysosomal membrane permeabilization (LMP), indicating physical or chemical insults tiny pores and compromise the integrity of the lysosomal limiting membrane, resulting in the controlled or catastrophic selective leakage of lysosomal contents into the cytosol (Yamashima, 2025). Therefore, LMP acts as either an initiator or a proteolytic amplifier that induces the final executioner phase of regulated necrosis (RN) (Alu et al., 2020).

Lysosome-dependent cell death (LDCD), regarded as a subclass of programmed cell death, has evolved from morphological observations to a mechanistically defined entity. LDCD is characterized by the translocation of cathepsins (cathepsins B, D, and L) and other hydrolases from the lysosomal lumen to the cytoplasm through the limited LMP, and they may engage diverse downstream execution pathways to activate apoptotic effects (Berg et al., 2022) and eventually cause cells death. HCC cells have malignant lysosome with changed structures and functions, which in turn makes cancer cells more sensitive to lysosomal instability (Domagala et al., 2018). Theoretically, LDCD can be used as a therapy to cause HCC cells dead. Indeed, LDCD has an excellent therapeutic effect on apoptosis-resistant and drug-resistant tumors (He et al., 2024). In contrast, autophagy, a way of apoptosis, which requires intact lysosomal function. LDCD is an ultimate consequence wherein lysosomal dysfunction becomes the cell death executioners itself. This distinction is particularly evident in cancer therapy (Wang Y. et al., 2023).

There are several factors that affect lysosomal membrane stability, including ROS-mediated damage, dysregulation of lysosomal pH, overloaded Ca^2+^ and disruptions in composition of sphingolipids and inhibition of heat shock protein 70 (HSP70) (Alu et al., 2020) which can trigger LMP and can be called as upstream mechanisms of LDCD (Zhang R. et al., 2025).

The downstream mechanisms of LDCD encompass three principal execution routes that collectively constitute a “death network” rather than isolated pathways. Firstly, the apoptotic route involves partial LMP, which releases cathepsin B (CTSB) into the cytosol. CTSB cleaves the pro-apoptotic Bcl-2 family protein Bid to generate truncated Bid (tBid), which activates Bax/Bak oligomerization, mitochondrial outer membrane permeabilization (MOMP), and cytochrome C release, thereby converging with the intrinsic apoptosis pathway (Kågedal et al., 2005). Secondly, the necrotic/necroptotic route engages activation of mixed lineage kinase domain-like pseudokinase (MLKL). The oligomerization of MLKL triggers LMP by aggregating and fusing lysosome. Then cathepsins sharply increase and cause necroptosis (Bi et al., 2025). Lastly, the ferroptotic route exploits the lysosome’s role as a major iron reservoir. LMP triggers ferroptosis (Alu et al., 2020) and releases ferrous iron (Fe^2+^) that catalyzes Fenton chemistry, generating reactive oxygen species (ROS) that propagate lipid peroxidation across cellular membranes and eventually leading to LDCD (Zhu et al., 2025). In a word, targeting LMP or LDCD to investigate new therapeutic strategies might be a promising field to HCC.

### 1.3 Natural products

Natural products (NPs) are bioactive compounds extracted from natural sources such as plants, animals, and microorganisms, characterized by abundant chemical diversity (Liu et al., 2025). Currently, NPs have garnered increasing attention as privileged scaffolds for inducing LDCD in cancer cells, owing to their multi-target engagement, structural diversity, wide-ranging sources, biological activities and favorable safety profiles compared to synthetic chemotherapeutics. Plant-sourced products and their derivatives like flavonoids, polyphenols, alkaloids, terpenes and saponins have proven anti-tumor properties (Zhang J. et al., 2024). NPs can “puncture” the hypertrophic lysosomes characteristic of HCC inducing LMP through ROS generation, pH neutralization, inhibition of lysosomal membrane repair mechanisms and modulation of multiple signaling pathways (Li et al., 2024b). HCC cells exhibit significant metabolic reprogramming in glycolysis, lipogenesis, and amino acid metabolism, providing a theoretical basis for targeting these metabolic pathways with natural products (Wang H. et al., 2021). Successfully, the potential of NPs serves as promising therapeutic agents that exploit lysosomal vulnerability in HCC.

This review aims to systematically dissect the molecular mechanisms of LDCD in HCC, categorize natural products according to chemical structure. Through integration, this paper is committed to constructing a strategic system for lysosome-targeted NPs, providing new ideas for breaking through the dilemma of drug resistance in liver cancer and improving outcomes in hepatocellular carcinoma.

## 2 Mechanisms of LDCD

**FIGURE 2:**
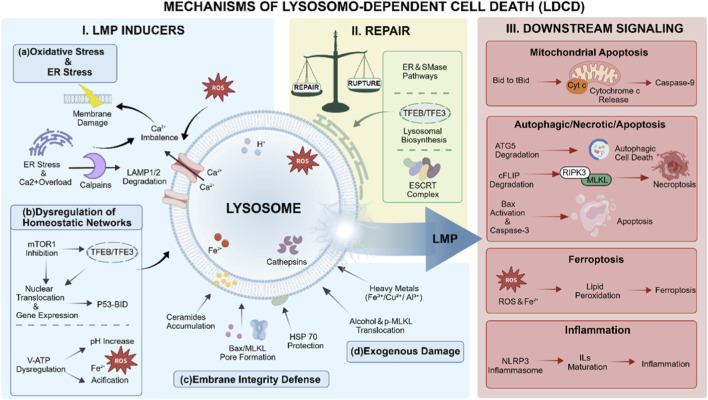
The mechanisms of LDCD. Supported by BioGDP (BioGDP - Generic Diagramming Platform for Biomedical Graphics).

LDCD is a form of cell death triggered by LMP, resulting in cellular damage caused by the leakage of lysosomal enzymes from lysosomes. LMP is triggered by various factors drives cell death through multiple pathways, including the release of lysosomal enzymes into the cytoplasm, cleavage of downstream substrates, activation of inflammatory pathways, and disruption of organelle homeostasis (Aits and Jäättelä, 2013) (Figure 2). Based on an integrated analysis of the LDCD mechanism, it involves the induction factors of LMP, cellular repair mechanisms, and downstream signaling pathways influencing cell death.

### 2.1 LMP

The pH in lysosomes is acidic, making digestive enzymes that can break down basic biological macromolecules such as proteins, lipids, and genetic material isolated inside lysosomes unable to play their role under normal circumstances. When the lysosomal membrane is damaged, these enzymes are released into the cytoplasm, potentially triggering cell death (Gemingnuer et al., 2025). Factors that contribute to LMP typically include oxidative stress imbalance disruption of lysosomal ion homeostasis, dysregulation of lysosomal homeostatic networks, membrane integrity defense system, exogenous damage. A deeper understanding of the factors that influence LMP can help find natural products that induce lysosome-dependent cell death.

#### 2.1.1 Oxidative stress imbalance

Oxidative stress is one of the key pathophysiological factors regulating LMP. Oxidative stress can be involved in all stages of cancer development, and it can promote pathological processes and tumor growth (Duan et al., 2024). When the rate of intracellular ROS generation exceeds the scavenging capacity of the antioxidant defense system, redox homeostasis is imbalanced, which may lead to disruption of the stability of lysosomal membrane structure. Specifically, abnormally elevated ROS levels can trigger intracellular calcium homeostasis dysregulation and induce calcium overload. This calcium overload can overactivate the transient receptor potential M2 channel (TRPM2), which promotes the outflow of large quantities of calcium ions stored in lysosomes, disturbs the local ionic environment, and ultimately triggers an increase in lysosomal membrane permeability. In addition, ROS can also directly attack biomembranes, modifying the phospholipid bilayer on the lysosomal membrane through lipid peroxidation reaction, and oxidizing the membrane integrin, resulting in the loss of membrane structural integrity and the release of hydrolytic enzymes in lysosomes into the cytoplasm. These released enzymes not only exacerbate organelle damage but also initiate cascade amplification effects. For example, it involves downstream cell death of mitochondria or the cascade effect of amplifying inflammation and aggravating damage (Gemingnuer et al., 2025). More importantly, the state of oxidative stress can activate specific signaling pathways and related enzymes, such as certain lipases or proteases, further positively regulating the LMP process, thereby amplifying the effects of lysosomal dysfunction at the molecular level and promoting cell death (Xiao et al., 2024; Gemingnuer et al., 2025). Oxidative stress is a key factor in the development of many human diseases, and natural products have the potential to treat various serious diseases by regulating the redox balance (Kim et al., 2023).

#### 2.1.2 Disruption of lysosomal ion homeostasis

As an important organelle, lysosomes can store and regulate calcium ions. The maintenance of calcium homeostasis constitutes a fundamental prerequisite for preserving the structural and functional integrity of the lysosomal membrane. The release of calcium from lysosomes can trigger diverse cellular responses, including cell death. Dysregulation of lysosomal Ca^2+^ homeostasis constitutes a critical disruption in intracellular ion balance, impacting lysosomal functionality. This imbalance may lead to the aberrant activation of calcium-dependent proteases, such as calpains, within the lysosomal compartment. The activation of these proteases can degrade key structural and regulatory proteins associated with the cells (Gemingnuer et al., 2025). Research by Pihán et al. (2021) reveals that RECS1 is a pH-sensitive calcium-permeable channel localized to the lysosomal membrane. This channel maintains lysosomal homeostasis by modulating acidification processes and intracellular calcium concentrations within lysosomes. Disturbances in these critical physiological parameters regulated by RECS1 activity can directly activate the intrinsic cell death pathways within the lysosomal compartment (Pihán et al., 2021). Endoplasmic reticulum stress can also induce elevated cytoplasmic calcium concentrations. This calcium surge stimulates calpain activation, which degrades lysosomal membrane proteins (e.g., LAMP1/2), leading to lysosomal rupture and ultimately lysosome-dependent cell death (Xiao et al., 2024).

#### 2.1.3 Dysregulation of lysosomal homeostatic networks

Transcription Factor EB (TFEB) functions as a master transcriptional regulator governing the coordinated expression of genes essential for lysosomal biogenesis and autophagic flux. Under normal physiological conditions, the activity of TFEB is controlled under physiological conditions by the mechanistic target of rapamycin complex 1 (mTORC1) signaling pathway, that TFEB is phosphorylated on its lysosomal surface and retained in the cytoplasm. When lysosomal function is impaired or nutrients are depleted, mTORC1 activity is inhibited, leading to TFEB dephosphorylation and nuclear translocation. The nuclear translocation of TFEB initiates transcription of target genes involved in lysosomal repair and autophagy clearance. Moreover, the activation of TFEB protects cells against damage and theoretically mitigates LMP effects. However, this compensatory mechanism frequently fails to adequately satisfy the heightened requirements of autophagy during the process of autophagy-dependent cell death (ADCD). Rather than effectively mitigating these demands, the response may instead result in an excessive burden being placed upon the lysosomal system. This overload consequently increases the risk of LMP and further compromises the overall stability of the lysosome (Gemingnuer et al., 2025; Wang X. et al., 2025). Once lysosomes are damaged, leaked lysosomal enzymes trigger downstream signaling pathways that may lead to LDCD.

MTORC1, a nutrient-sensing complex located on the cytoplasmic side of lysosomes, regulates both TFEB and lysosomal integrity. Under nutrient-sufficient conditions, mTORC1 suppresses autophagy and maintains lysosomal homeostasis. During stress or nutrient deprivation, the reduced activity of mTORC1 renders cells more susceptible to LMPs. In addition, The V-ATPase regulates mTORC1 activity and maintains lysosomal acid pH. Inhibition or dysfunction of the V-ATPase leads to reduced lysosomal acidification, which not only affects protease maturation but also disrupts membrane stability, making lysosomes more susceptible to changes in permeability (Aits and Jäättelä, 2013).

Disruption of lysosomal pH homeostasis is an important pathological event underlying LDCD. The V-ATPase (a multi-subunit complex located on lysosomal membranes) regulates mTORC1 activity and maintains lysosomal acid pH. Inhibition or dysfunction of the V-ATPase leads to reduced lysosomal acidification, which not only affects protease maturation but also disrupts membrane stability, making lysosomes more susceptible to changes in permeability (Aits and Jäättelä, 2013). Research by Ren et al. (2025) reveals that Transcriptional Factor 25 (TCF25), as a key nutrient-sensing molecule, can transfer to the lysosomal surface to bind to V-ATPase under prolonged glucose deprivation, adding more protons to the lysosome. Acidic enhancement in lysosomes causes NCOA4 to transport ferritin to lysosomes for degradation, which continues. This leads to Fe^2+^ accumulation within lysosomes, triggering the Fenton reaction to generate substantial ROS, ultimately inducing LMP and LDCD (Ren et al., 2025). This suggests that extremely high or low pH levels in lysosomes may induce LMP through different mechanisms.

In some metabolic diseases, when alkaline metabolites accumulate abnormally in lysosomes, neutralizing their acidic environment will lead to an increase in pH. The changes of pH can inactivate pH-sensitive enzymes such as cathepsin and cause accumulation of undegraded substrates, including misfolded proteins and lipids, in lysosomes. These raw substrates in turn trigger mitochondrial dysfunction and amplify the transmission of cell death signals. These undigested substrates subsequently trigger mitochondrial dysfunction and amplify cell death signaling pathways (Wang X. et al., 2025).

P53, a protein encoded by the TP53 gene, activates upon exposure to DNA-damaging agents. P53 induces LMP via the p53-BID axis while simultaneously inhibiting LMP through the p53-mTOR-TFEB/TFE3 pathway (Yamashita et al., 2022). The mutations of p53 are common in HCC, and the mTOR pathway is frequently overactivated (Liu Y. et al., 2024), rendering lysosomes more sensitive to LMP-inducing factors.

#### 2.1.4 Membrane integrity defense system

The integrity of lysosomal membrane is regulated by multiple molecules. The processes of lipid metabolism localized on the membrane play a dual role in regulating membrane stability, both promoting damage and mediating protective effects. Taking ceramides as an example, this molecule is a core bioactive lipid in the sphingolipid metabolic pathway, and its production mainly depends on the hydrolysis of sphingomyelinase on sphingomyelin (Spiegel and Merrill, 1996). Under specific stress conditions, the accumulation of ceramides can induce an increase in LMP, as the formation of sphingosine leads to the release of lysosomal hydrolases and other intraluminal components into the cytoplasm, lysosomal hydrolases will activate the downstream cell death signaling pathway, promoting the occurrence and development of LDCD. In contrast, another important metabolite, sphingosine-1-phosphate (S1P), exhibits the opposite biological effect (Gemingnuer et al., 2025). S1P is produced by phosphorylation of sphingosine catalyzed by sphingosine kinase, which can initiate anti-apoptotic signal transduction pathways in cells, enhance cell viability (Takuwa et al., 2012) and provide protective support for lysosomal membrane structure (Xiao et al., 2023).

Proteins play a critical role in maintaining the structural stability of cellular membranes. These macromolecules are actively involved in the regulation of membrane integrity through diverse molecular mechanisms. B-cell lymphoma 2 (Bcl-2) family proteins maintain lysosomal membrane integrity through direct interaction, while heat HSP70protects membrane structure from stress damage via its molecular chaperone function (Wang X. et al., 2025). Conversely, upon activation, pro-apoptotic proteins Bcl-2-associated X protein (BAX) and MLKL translocate to the lysosomal membrane, forming pores through protein-protein interactions that directly induce LMP (Dong H. et al., 2024). Additionally, extracellular death ligands such as tumor necrosis factor-alpha (TNF-α) and Fas ligand (FasL) can bind to specific receptors on the cell surface. This interaction activates a downstream signaling pathway that leads to the disruption of lysosomal membrane architecture and the subsequent formation of pores within the membrane (Gemingnuer et al., 2025).

Researcher finds that the LCDR-hnRNP K-LAPTM5 axis maintains lysosomal membrane integrity. Furthermore, knockdown of any component on this axis (LCDR, hnRNP K, or LAPTM5) increases LMP, suggesting this signaling axis plays a critical role in maintaining lysosomal homeostasis (Yang X. et al., 2022).

#### 2.1.5 Exogenous damage

In addition to endogenous factors, a variety of exogenous stressors can directly or indirectly induce LMP, including metal ions, specific compounds, and alcohols. For example, metal ions such as Fe^3+^, Cu^2+^, and Al^3+^ can catalyze local oxidation reactions that damage lysosomal membranes from within. In addition, physical stress or certain lysosomal tropism reagents, which can accumulate in lysosomes and disrupt their membrane integrity, may also cause physical damage to the lysosomal membrane (Gemingnuer et al., 2025). Alcohol can induce phosphorylation modification of the mixed lineage kinase-like domain protein (MLKL). This modification led to the transposition of phosphorylated forms of MLKL (P-MLKL) to the lysosomal membrane and a significant increase in LMP (Jin et al., 2025).

### 2.2 Cellular repair mechanisms

As a key factor in cell metabolism, lysosomes affect degradation, recovery, metabolic regulation and other functions. Following lysosomal damage, repair factors are recruited to initiate repair mechanisms. Furthermore, the function and quantity of lysosomes are regulated by multiple mechanisms. For example, TFEB and TFE3 are the core transcription factors that regulate lysosomal number and function (Sardiello et al., 2009). Multiple pathways activate transcription factor TFEB/TFE3, thereby promoting lysosomal biosynthesis following injury. For example, due to lysosomal damage, calcium ion efflux activates calcineurin (Wróbel et al., 2022), inhibition of mTORC1 activity (Yang et al., 2018), and changes in the lipid environment (Zhitomirsky et al., 2018) may activate TFEB/TFE3. These are all methods of phosphorylation. In addition to phosphorylation, the activity of TFEB/TFE3 is also regulated by acetylation, oxidation, SUMOylation, ubiquitination, etc. Calcium signaling also serves as a critical trigger for lysosomal membrane repair. Lysosomal membrane damage induces calcium ion efflux, recruiting membrane repair complexes such as ESCRT to counteract LDCD and promote cell survival. Additional repair pathways mediated by the endoplasmic reticulum (ER) and sphingomyelinase (SMase) exist. They can both be repaired by affecting the properties of lysosomal membranes (Radulovic et al., 2026). In addition, when cells are under stress or experience nutrient deprivation, the dissociation of Bromodomain Protein BRD4 lifts the repression of autophagy and lysosomal genes. Previously repressed lysosomal genes (such as LAMP1 and cathepsins) begin to be transcribed and expressed at high levels. The production of more lysosomes enhances autophagy, provides nutrients, and helps cells survive (Sakamaki et al., 2017). When exploring natural products that promote lysosomal rupture and enzyme release to induce lysosome-dependent cell death, attention should also be paid to repair mechanisms. Targeting these repair pathways may enhance pro-apoptotic effects.

### 2.3 Downstream signaling

#### 2.3.1 Mitochondrial apoptosis pathway

Mitochondrial Apoptosis Pathway represents the core execution pathway of LDCD. Upon release from lysosomes, cathepsins induce cell death through multiple mechanisms. Cathepsins cleave the pro-apoptotic Bcl-2 family member Bid, generating tBid. TBid subsequently transfers to the mitochondrial outer membrane, inducing outer membrane permeabilization. This leads to cytochrome c release and activation of the apoptosome complex, ultimately initiating caspase-9-dependent apoptotic execution (Wang X. et al., 2025). Furthermore, cathepsin D directly processes caspase-8, while cathepsin L degrades autophagy-associated proteins such as autophagy-related protein 5 (ATG5), thereby directing cellular fate toward autophagic cell death (Aits and Jäättelä, 2013). Notably, the pathway of cathepsin directly acting on proteins bypasses the direct mitochondrial protection provided by anti-apoptotic proteins like Bcl-2. Thus, we can infer that effective induction of cell death remains possible even in Bcl-2-overexpressing, apoptosis-resistant cancer cells.

#### 2.3.2 Cell death-related pathways

In the absence of caspase activity, cathepsins degrade anti-apoptotic factors like cFLIP, thereby promoting necrotic apoptosis through RIPK3/MLKL activation (Aits and Jäättelä, 2013). In addition to degrading anti-apoptotic factors, they can induce apoptosis in two ways: by activating pro-apoptotic factors (such as BAX) or by directly cleaving caspase-3.

Under pathological conditions, lysosomal membrane damage leads to massive zinc efflux via upregulated TRPML1 channels, which directly activate necrotic cell death pathways. Abnormal release of lysosomal iron stores triggers a cascade of harmful reactions: released iron catalyzes Fenton reactions and lipid peroxidation, significantly elevating intracellular ROS levels. This elevated ROS not only exacerbates oxidative damage but may also accelerate ferroptosis (Wang X. et al., 2025). It involves NCOA4-mediated ferritin autophagy (Ren et al., 2025), which leads to excessive iron accumulation in lysosomes, resulting in LMP and thereby amplifying ferroptosis.

#### 2.3.3 Amplification of inflammatory pathways

The pathological effects of lysosomal enzyme leakage extend beyond autonomous cell death, serving as a critical trigger for inflammatory cascades. For example, the leakage of cathepsin B activates the NLRP3 inflammasome by promoting the release of mitochondrial damage-associated molecular patterns and potassium efflux, leading to facilitating the proteolytic conversion of pro-cystatin-1 to its active form, which then cleaves pro-interleukins into mature cytokine forms for extracellular secretion (Chevriaux et al., 2020). This amplifies inflammatory pathways, potentially generating inflammation or exacerbating existing inflammation. This is also a factor that needs to be considered while inducing the release of cathepsin from LDCD.

The important role of lysosomal enzymes in activating cell death and inflammation makes them a key regulatory node in LDCD-related diseases. In HCC, various forms of programmed cell death do not operate independently; rather, they form a complex, interconnected network (Chen L. et al., 2024). LDCD is characterized by triggering multiple pathways downstream of LMP, involving various modes of cell death. Focusing on the lysosomal characteristics of hepatocellular carcinoma, it is possible to induce hepatocellular carcinoma cells to enter the LDCD. Combined with downstream signals of LDCD, if hepatocellular carcinoma cells block a single cell death pathway through mutations or dysregulation, other pathways may still lead to their death. Alternatively, inducing cancer cells to undergo multiple forms of cell death may enhance the efficacy of anticancer therapy.

## 3 Lysosomal characteristics of HCC

### 3.1 Metabolic abnormalities and reduced membrane stability

Compared with normal cells, HCC cells have increased oxidative stress (Yuan et al., 2025), and high-speed metabolism to produce ROS and abnormal lipids, causing their lysosomal membranes to be in a state of high pressure and membrane stability to decrease. This state may make the lysosomes of HCC more vulnerable to LMP, which can eventually lead to LDCD.

High concentrations of ROS have been shown to induce HCC cell death (Geng et al., 2017; Yuan et al., 2025). HCC cells maintain high ROS levels while concurrently upregulating antioxidant mechanisms, including the Nrf2 pathway and GSH system, to sustain redox homeostasis and drive proliferation. In particular, Nrf2 is a key nuclear transcription factor regulating the cellular redox balance. Activation of the Nrf2 pathway can alleviate oxidative stress and lipid accumulation (Liu et al., 2020; Zhang Y. P. et al., 2021). Low concentrations of ROS induce the expression of epithelial-mesenchymal transition (EMT) and matrix metalloproteinases (MMPs), enhancing the invasiveness. Besides, ROS can also activate PI3K/AKT/mTOR and MAPK/ERK pathways to promote tumor growth (Yuan et al., 2025). Thus, HCC itself has high ROS levels, and the upregulation of its antioxidant system changes the environment around lysosomes. For the treatment of HCC to varying degrees, antioxidants can be used to reduce ROS in the early stage, and ROS-induced cancer cell death can be further increased in the advanced stage. Besides, in HCC, researchers find the metabolic stress of lipid overload in lysosomes in HCC. Wang et al.'s study finds that the level of sphingomyelin in cancer cells is significantly higher than that of non-cancer cells, which changed the composition of lysosomal membranes, leading to an increase in lysosomal volume. The reduced membrane stability of hypertrophied lysosomes is more likely to occur LMP (Wang Y. et al., 2023). Lipid peroxidation caused by high levels of ROS in HCC cells is also one of the reasons for lysosomal membrane instability and easy LMP to occur (Aits and Jäättelä, 2013; Xiao et al., 2024). Yagi et al. (2023) showed that the absence of the expression of the mitochondrial serine transporter SFXN1 in non-viral HCC may reduce iron ion transport to mitochondria, reduce ROS production and ferroptosis, and SFXN1 deletion may help tumor cells survive under metabolic stress of lipid overload. Ceramide levels in HCC tissues are significantly reduced compared to normal liver tissues. Increasing Ceramide levels at tumor sites is considered a viable therapeutic strategy due to its involvement in various signals such as lysosomal permeability and promoting cancer cell death. HCC cells become resistant to drug therapy due to upregulation of glucoceramide synthase (GCS) to reduce the pro-apoptotic effects of ceramide, an enzyme encoded by the SMPD1 gene, which is responsible for hydrolyzing sphingomyelin (SM) into ceramide and phosphocholine. In the liver, ASMase mediates TNF-α/Fas-induced apoptosis of hepatocytes. However, the levels of ceramide in HCC tissues are usually low, and ASMase interacts with autophagy and mTOR pathways, and the levels of ASMase are often reduced in HCC, which is conducive to the growth of cancer cells (Mir and Thirunavukkarasu, 2023).

C-Jun N-terminal kinase (JNK) maintains lysosomal homeostasis in HCC cells by stabilizing LAMP2A, a lysosomal membrane protein. The study by Desideri and Ciriolo (2021) demonstrates that the regulation of LAMP2A by JNK is cancer-specific: inhibition of JNK in HCC cells leads to a decrease in LAMP2A levels. Interestingly, inhibition of JNK in normal hepatocytes leads to an increase in LAMP2A levels. Activation of the JNK pathway is also the cause of chemotherapy resistance in hepatocellular carcinoma. When JNK is inhibited in hepatocellular carcinoma cells, the reduction of LAMP2A will exacerbate lysosomal damage caused by lysosomal targeted drugs, ultimately leading to cell death (Desideri and Ciriolo, 2021). Moreover, oxidative stress alters the expression of LAMP2A, and JNK can maintain the stability of LAMP2A, suggesting that JNK may play a buffering role in oxidative stress.

Oxidative stress and lipid peroxidation in hepatocellular carcinoma cells alter the membrane stability and integrity of lysosome. In this case, the lysosomes of HCC may be in a state of high pressure and high load, which is easily affected by external factors.

### 3.2 Core functional molecular characteristics

In LDCD, the core molecule that performs cell death is cathepsin in lysosomes. HCC cells will express high cathepsin for survival, but once LMP occurs, cathepsin leaks and the death signal will be abnormally amplified.

Cathepsins, predominantly localized within lysosomes, have been implicated in the promotion of HCC cell growth. CTSV is cathepsin V in the lysosome of HCC, and Wang Z. et al. (2024)’s study confirms that knocking down the CTSV gene *in vitro* can reduce the proliferation and invasion of HCC cell lines, which can promote the growth and metastasis of HCC cells. Through molecular docking to screen natural small molecules including Procyanidin A2 (proanthocyanidin A2), Isocrenatoside and so on, which are predicted to bind to the active site of the CTSV protein and may block its function. CTSB and CTSL are also overexpressed in HCC cells, both of which can promote cell growth and proliferation. Cathepsin (CTSB, CTSL, CTSS) activates the PI3K/Akt/mTOR signaling pathway through phosphorylation of Akt to promote the uncontrolled growth and proliferation of HCC cells. Results show that drugs can inhibit the maturation of lysosomal PH and cathepsin, thereby fighting HCC (Xue and Liu, 2021; Yu H. et al., 2023).

When cathepsin is released into the cytoplasm, it drives apoptosis. TNF-α or TRAIL causes lysosomal membrane instability, triggers LMP, and cathepsin (CTSB, CTSD, CTSL) is released into the cytoplasm, cleaving the Bid protein and ultimately leading to apoptosis downstream of the LDCD mechanism (Aits and Jäättelä, 2013; van Mourik et al., 2022; Wang X. et al., 2025). In addition, cathepsin is secreted outside the cell, which increases VEGF levels, activates MMPs and uPAs (matrix-degrading proteases), and cuts the extracellular matrix (ECM), resulting in cancer cell metastasis and vascular formation (van Mourik et al., 2022). HCC tissue is characterized by abundant blood vessels and abnormal structure and function.

In addition, recent studies have found that there is also an enzyme that is highly expressed in HCC cells. ARL8B, which is mainly involved in the regulation of lysosomal movement and function, regulates the distribution, morphology and acidic environment of lysosomes. Tumor cells with high ARL8B expression appear more vulnerable because they are highly dependent on this pathway. Wu L. et al. (2025)’s study has confirmed that patients with high ARL8B expression respond better to drugs such as sorafenib. Furthermore, IGF-1 upregulates cathepsin activity, and these proteases—particularly CTSB—promote HCC cell proliferation, migration, and invasion, thereby mediating the pro-tumorigenic effects of IGF-1 (van Mourik et al., 2022).

### 3.3 Abnormal pathway regulation

To maintain an abnormally large lysosomal system, cancer cells are highly dependent on specific regulatory pathways to maintain homeostasis, which not only regulate cancer cell survival but also affect the sensitivity of HCC to LDCD.

The progression of cancer cells is promoted by cell signaling, which largely depends on the secretion of soluble molecules and the exchange of extracellular vesicles (EVs). Malignant cells usually secrete more exosomes due to oxidative stress promoting TFEB, and β-hexosaminidase B (HEXB) is involved in mediating oxidative stress-induced release of EV, thereby promoting the development of HCC. Knockdown of HEXB or the use of its inhibitors can significantly inhibit the growth of HCC, which has been confirmed by Gemingnuer et al., (2025). The core survival-promoting pathway of HCC is the PI3K/Akt/mTOR signaling pathway, which is also a more classical pathway that can be activated by cathepsin (van Mourik et al., 2022). Liu Y. et al. (2024)’s study confirms that overactivation of the reprogrammed branched-chain amino acid (BCAA) mTOR signaling pathway in TP53-mutant hepatocellular carcinoma showed higher sensitivity to mTOR inhibitors and that ROS and p53 mutually promote each other’s production and synergistically induce hepatocellular cell death (Geng et al., 2017). SIRT1 is upregulated in hepatocellular carcinoma cells, which has pro-tumor effects. However, before nonalcoholic steatohepatitis (NASH) transforms into HCC, SIRT1 usually has an inhibitory effect on the inflammatory regulator NF-κB. However, SIRT1 is inhibited by CTSB in HCC, and NF-κB is activated, which in turn triggers inflammation (van Mourik et al., 2022). The shift in the role of SIRT1 may be due to the high expression of CTSB in hepatocellular carcinoma cells. A study by Dai et al. (2023) reveals that NLRP3 is downregulated in HCC cells. The combined activation of lipopolysaccharide (LPS) and adenosine triphosphate (ATP) can activate the NF-κB signaling pathway, thereby inhibiting the proliferation and migration of HCC cells. This suggests that targeting the activation of the NLRP3 inflammasome may represent a potential strategy for treating HCC.

Tumor cells have an increased demand for protein folding, resulting in endoplasmic reticulum stress, which leads to cell death. However, in this case, lncRNAs (long non-coding RNAs) that promote cell death, such as GAS5, MEG3, RMRP, and NBR2, are commonly inhibited or downregulated in HCC cells. The lncRNAs that inhibit cell death are MALAT1, GOLGA2P10, and HOTAIR/ZFAS1, which tend to be activated. Current research may overcome drug resistance in HCC by delivering or upregulating MEG3 or restoring the expression of pro-death oncogenes such as GAS5 by pushing endoplasmic reticulum stress towards the “death pathway” (Goyal et al., 2024).

The disorders and abnormal deposition of iron and copper metabolism in the liver are important factors in promoting the occurrence and development of HCC. Iron-copper overload will lead to oxidative stress and DNA damage. Thus, under normal circumstances, inducing ferroptosis or copper death can kill cancer cells. However, in the early stages of HCC occurrence and development, iron and copper can cause a large number of cell death and inflammation, and they can enhance the proliferation and metabolic activity of HCC cells, promote autophagosome formation, and help cancer cells survive. Moreover, the long-term inflammatory repair process promotes the formation of cirrhosis and HCC. The abundant presence of iron and copper allows lipid peroxidation and the formation of autophagosomes in large quantities. This phenomenon puts lysosomes under high pressure, which may reduce lysosomal membrane stability (Chatzikalil et al., 2025; Pang, 2025). GPX4 is a key enzyme involved in the suppression of ferroptosis, and study has shown that restoring GPX4 activity can protect tissues from damage (Zeng et al., 2023). Therefore, simultaneously targeting lysosomes and inhibiting GPX4 may be an effective strategy for inducing synergistic cell death in HCC.

## 4 Natural product-induced LDCD in HCC

**FIGURE 3:**
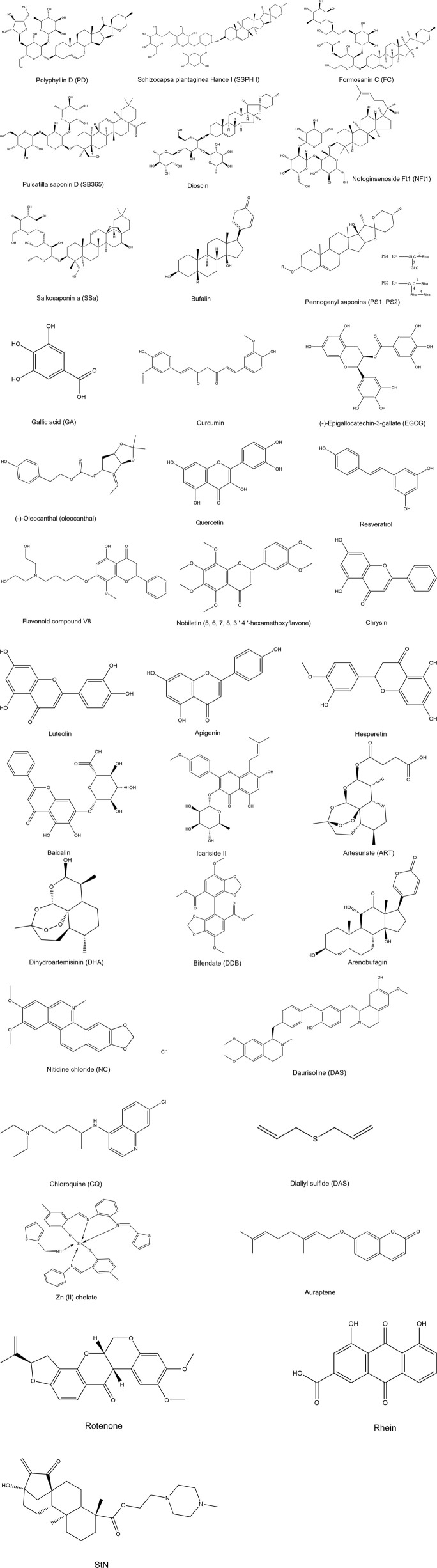
The chemical structures of natural products targeting LDCD or LMP in HCC. Supported by BioGDP (BioGDP - Generic Diagramming Platform for Biomedical Graphics).

NPs play an important role in inducing LDCD, with structural diversity (Figure 3), multi-target effect and good safety. These compounds take advantage of the unique vulnerability of malignant lysosomes, particularly the “hypertrophic” phenotype of increased size, elevated iron content, and altered membrane composition to converge on LMP and subsequent cell death through different mechanisms, achieving the effect of suppressing liver cancer. We have classified these natural products according to different types (Table 1), elucidate the key targets, regulatory directions, and downstream effects of each compound, providing a systematic reference for the subsequent development of LDCD-based natural product HCC treatment strategies.

**TABLE 1:** Natural products inhibit HCC through regulating LDCD/LMP.

Class	Compound	Sources	Target/Pathway regulation	Effect	References
Steroidal saponins	Polyphyllin D (PD)	*Paris polyphylla* rhizome	↓ SMPD1	Sphingomyelin accumulation	Wang Y. et al. (2023)
	*Schizocapsa plantaginea* Hance I (SSPH I)	Schizocapsa plantaginea rhizome	↓ NAC and U0126	Blocking autophagosome–lysosome fusion and inducing ROS	Zhou et al. (2024)
			↑ MAPK/ERK1/2 signaling pathway	Lysosomal degradation	
			↓ LAMP1 and LAMP2A		
			↑ SLC7A5	Triggering ferroptosis	Pandey et al. (2024)
			↑ TFR and Fpn proteins		
	Formosanin C (FC)	*Paris polyphylla*	↑ LC3-II	Triggering ferroptosis and inducing ROS	Pandey et al. (2024)
			↓ GPX4		Rodriguez et al. (2024)
	Pulsatilla saponin D (SB365)	Pulsatilla chinensis (*P. chinensis,* Bai Tou Weng)	↑ p62 level and LC3-II/I ratio	Inhibiting autophagic flux	Hong et al. (2019)
	Notoginsenoside Ft1 (NFt1)	*Panax notoginseng*	↑ CTSB, CTSD, LAMP1, LAMP2, and TPP1	Sensitive to LMP	Ferret et al. (2025), Jeon et al. (2025)
			↓ PI3K/AKT/mTOR pathway		
			↑ TFEB		
	Dioscin	Fenugreek	↓ PI3K/Akt/mTOR pathway	Inducing ROS and LMP	Zhang et al. (2018)
			↑ JNK		
	Bufalin	Posterior auricular glands and the skin of *Bufo gargarizans*	↓ PI3K/AKT/mTOR pathway	LMP	Xie et al. (2021)
	Saikosaponin a (SSa)	*B. falcatum*	↓ Bcl-2	Reduce the lysosomal membrane stability	Tu et al. (2019)
			↑ BAK, BAD, PUMA		
	Pennogenyl saponins (PS1, PS2)	Rhizoma Paridis	↑ BAX, ↓ Bcl-2	LMP	Long et al. (2015)
			↑ cytosolic caspase-9 and caspase-3		
			↑ p38 and JNK		
			↓ PI3K/Akt signaling pathway		
Polyphenols	Curcumin	The rhizome of turmeric	↑ ROS	LMP	Gemingnuer et al. (2025)
			↑ AMPK-JNK pathway		
			↑ TFEB		
	Gallic acid (GA)	Various fruits, plants and nuts	↓ SLC7A11 and GPX4	Triggering ferroptosis and inducing ROS	Al Amin et al. (2025)
	(-)-Epigallocatechin-3-gallate (EGCG)	Green tea	↑ ROS	LMP	Halaby (2021)
	(-)-Oleocanthal (oleocanthal)	Extra virgin olive oil oleocanthal	↑ ROS	LMP sphingomyelin accumulation	Goren et al. (2019)
			↓ ASM		LeGendre et al. (2015)
Flavonoids	Quercetin	Fruits, vegetables, and grains	↑ ROS	Triggering ferroptosis	Tuli et al. (2023)
			↑ TFEB		
			↑ pH	LMP	Gemingnuer et al. (2025)
	Nobiletin (5, 6, 7, 8, 3′4′-hexamethoxyflavone)	Zhishi (Rutaceae) juice	↓ Bcl-2 and COX-2	LMP	Ma et al. (2014)
			↑ Bax and caspase-3		
	Flavonoid compound V8	—	↓ HSP70-BMP interaction	Sphingomyelin accumulation	Chen et al. (2025)
			↓ TRPML 1, PPP3CB and TFEB	LMP	
	Resveratrol	Grapes and berries	↓ v-ATPase	Increasing lysosomal pH and LMP	Gemingnuer et al. (2025)
	Chrysin	Oroxylum indicum (L.)Vent, Pinus mon-ticola Dougl et al.	↓ Bcl-2 and ↑ Bax	LMP	Yang et al. (2023)
	Luteolin	Chrysanthemum morifolium, *apium graveolens*, thymus vulgaris et al.	↓ Bcl-2 and ↑ Bax	LMP	Yang et al. (2023)
	Apigenin	Chamomile, parsley, celery et al.	↓ Bcl-2 and ↑ Bax	LMP	Yang et al. (2023)
			↓ PI3K/AKT pathway		
			↑ ROS		
	Hesperetin	Citrus fruits	↓ Bcl-2 and ↑ Bax	LMP	Yang et al. (2023)
	Baicalin	Scutellaria baicalensis	↑ pH	LMP	Dong X. et al. (2024)
	Icariside II	Epimedium Linn	↓ LAMP-1	Lysosomal degradation	Halaby (2021)
Artemisinin derivatives	Artesunate (ART)	Semisynthetic derivative of the antimalarial artemisinin	↑ CTSB/CTSL	Triggering ferroptosis and inducing ROS	Li et al. (2021)
			↓ SLC7A11		
			↓ GPX4		Rodriguez et al. (2024)
	Dihydroartemisinin (DHA)	Semisynthetic derivative of the antimalarial artemisinin	↑ PEBP1/15-LO	Triggering ferroptosis and inducing ROS	Su et al. (2021)
Esters	Bifendate (DDB)	Schisandrae chinensis	↑ LC3-II and p62	Inhibiting autophagy	Yuan et al. (2022)
			↓ ATG5		
			↓ SNAP29-STX17-VAMP8 SNARE complex formation		
	Ent-13-Hydroxy-15-kaurene-19-acid N-Methylpiperazine Ethyl Ester	Stevia rebaudiana (Bertoni)	↓ lysosomal CTSB	DNA damage	Guo et al. (2024)
	Arenobufagin	Toad venom	↓ PI3K/AKT/mTOR pathway	LMP	Liu B. et al. (2024)
Alkaloid	Nitidine chloride (NC)	Zanthoxylum nitidum (Roxb.)	↓ mTORC1	Lysosomal degradation	Chen F. et al. (2024)
			↑ ATF4 and Sestrin2		
			↓ IGF2R		
	Daurisoline (DAS)	Rhizoma Menispermi	↑ LC3-II and p62	Inhibiting autophagic flux	Xue and Liu (2021)
			↑ pH	LMP	
	Echium amoenum	*Alstonia scholaris*	↑ ROS	LMP	Zarei et al. (2023)
Organosulfur compound	Diallyl sulfide (DAS)	Garlic	↑ p-AMPK	Inhibiting autophagic flux	Yu H. et al. (2023)
			↓ p-mTOR		
			↑ pH	LMP	
			↓ LAMP1 and cathepsin D		
Coumarin	Auraptene	*Citrus aurantium* and *Aegle marmelos*	↑ ROS	LMP	Li et al. (2024a)
			↓ SLC7A11	Triggering ferroptosis	
Others	Zn (II) chelate	—	↑ ROS	LMP	Mousa et al. (2025)
	Chloroquine (CQ)	—	↑ pH ↓ ASM	LMP sphingomyelin accumulation	Piao and Amaravadi (2016)
	Rotenone	Roots and stems of Derris, Tephrosia, Lonchocarpus and Mundulea plant species	↑ ROS	LMP	Wei et al. (2025)
			↑ pH		
			↓ lysosomal m-CTSB, m-CTSD, m-CTSL		
			↓ LAMP1	lysosomal degradation	
	Rhein	The dried root of the Polygonum family palm-leaved rhubarb	↓ HSP72/HSC70/GRP78	LMP	Wang J. et al. (2024)

### 4.1 Steroidal saponins

Saponins are found in a variety of higher plants and exhibit the most comprehensive range of functions, covering five major areas in triggering LMP and LDCD: inhibitory effects, activating effects, ROS regulation, cell death regulation, and autophagy regulation, making it a typical example of a multi-target agent.

Polyphyllin D (PD) is a steroid saponin separated from the *Paris polyphylla* rhizome, showing anticancer effects on HCC. Wang identified PD can induce LMP by interacting with the Trp148 site of sphingomyelinase SMPD1. SMPD1 inhibition disrupts the balance and causes sphingomyelin accumulation, reduced membrane fluidity, and LMP in hepatocellular carcinoma cells. Moreover, PD also enhances the anti-cancer efficacy of sorafenib by directly targeting hypertrophic lysosomes to disrupt lysosomotropism and release trapped sorafenib (Wang Y. et al., 2023).

Saponin of *Schizocapsa plantaginea* Hance I (SSPH I), a novel bioactive phytochemical isolated from the rhizome of Schizocapsa plantaginea, shows ability to block autophagosome–lysosome fusion by targeting NAC (ROS inhibitor) and U0126 (MEK1/2 inhibitor). Therefore, SSPH I induces ROS and activates MAPK/ERK1/2 signaling pathway, leading to the decrease of the expression of lysosomal membrane proteins (LAMP1 and LAMP2A), which causes lysosomal degradation (Zhou et al., 2020). Moreover, SSPH I improves the expression of SLC7A5 and increases the levels of TFR and Fpn proteins, leading to the accumulation of Fe^2+^. Then iron overload triggers ferroptosis in HCC (Pandey et al., 2024).

Formosanin C (FC), a diosgenin saponin from *P. polyphylla*, induces LDCD in HCC by the lysosome-iron-ferroptosis axis. FC has the capacity to promote ferritinophagy-mediated iron release and lipid ROS production. FC induces autophagic flux through inhibiting the degradation of LC3-II and triggers ferroptosis (Pandey et al., 2024). The released ferrous iron catalyzes the Fenton reaction, producing ROS which trigger membrane damage and inactivate the core defense enzyme GPX4 of ferroptosis (Rodriguez et al., 2024).

Pulsatilla chinensis (*P. chinensis*, Bai Tou Weng) is full of triterpenoid saponins. Among those, pulsatilla saponin D (SB365) significantly inhibit tumor growth in HCC by suppressing autophagic flux and increasing the lysosomal pH (Hong et al., 2019). The p62 level and LC3-II/I ratio is observed increase in a time-dependent manner, indicating that SB365 inhibits autophagic flux, and membrane stability decreases, leading to LMP. Subsequently, LMP causes leakage of cathepsin B and/or cathepsin D from the lysosome into the cytoplasm and increase of intracellular ROS levels, and ultimately triggers LDCD (Hong et al., 2019).

Notoginsenoside Ft1 (NFt1), a bioactive saponin from *Panax notoginseng*, treat HCC through the mechanism that NPs regulate lysosomal biogenesis and cell death through transcriptional regulation. NFt1 upregulates genes associated with lysosomal cell death (CTSB, CTSD, LAMP1, LAMP2, and TPP1), which are related to the ability to inhibit the PI3K/AKT/mTOR pathway. The inhibition of the PI3K/AKT/mTOR pathway can remove phosphorylation inhibition of TFEB and enhance TFEB activity (Jeon et al., 2025). TFEB enhances lysosomal biogenesis and autophagy flow, causing lysosomes to load a large number of substrates to be degraded, thereby making cells more sensitive to subsequent LMP triggers (Ferret et al., 2025).

Dioscin, a steroidal saponin separated from fenugreek (Li et al., 2010), inhibits the PI3K/Akt/mTOR pathway. Moreover, dioscin increases intracellular ROS generation and the mRNA expression of JNK gene (Zhang et al., 2018).

Bufalin, as a cardiac steroid extracted from posterior auricular glands and the skin of *Bufo gargarizans*, is proven to perform anticancer activity. Bufalin inhibits the transfer and invasion of HCC by PI3K/Akt/mTOR signaling pathway to decrease mTOR signal activation and further causes LMP (Xie et al., 2021).

Saikosaponin a (SSa), major active component of triterpene saponins in *B. falcatum*, shows ability to decrease expression of Bcl-2 and increase expressions of BAK, BAD, p53-upregulated modulator of apoptosis (PUMA) and translocation of BAX and BAD. These changes reduce the stability of the lysosomal membrane and release apoptotic factors (Tu et al., 2019).

Pennogenyl saponins are characteristic components of Rhizoma Paridis, among which, only PS1 and PS2 can selectively inhibit HCC growth. PS1 and PS2 increase pro-apoptotic protein Bax and decrease anti-apoptotic protein Bcl-2, and subsequently activate cytosolic caspase-9 and caspase-3. Therefore, PS1 and PS2 lead to BAX metastasis inducing LMP. PS1 and PS2 also increase the phosphorylation of p38 and JNK while inhibiting the PI3K/Akt signaling pathway in HepG2 cells to trigger and exacerbate LMP (Long et al., 2015).

### 4.2 Polyphenols

Polyphenols are a large group of plant-derived secondary metabolites that contain multiple phenol -hydroxyl groups, have diverse structures, and have antioxidant activity as the core activity, which functionally focused on ROS-related regulation and metabolic pathway regulation (e.g., AMPK/mTOR pathway).

Curcumin is a natural polyphenol extracted from the rhizome of turmeric (Rainey et al., 2020). Its mechanism of action involves the induced production of ROS, which in turn triggers lipid peroxidation reactions, ultimately leading to LMP, disruption of lysosomal structure, release of cathepsins (Gemingnuer et al., 2025) and eventually cancer cells death. Curcumin is also proved as TFEB activators to mediate lysosomal activation. It works as activating AMPK-JNK pathway so to suppress mTOR (Gemingnuer et al., 2025). Activated TFEB is more likely to induce cancer cells to LMP.

Gallic acid (GA) and its derivatives are polyphenols with anticancer effects. GA can reduce ferroptosis-related proteins SLC7A11 and GPX4 activity, increase ROS generation and lipid peroxidation, causing membrane damage (Al Amin et al., 2025).

(-)-Epigallocatechin-3-gallate (EGCG) is the most extensive studied tea polyphenol for its anti-cancer function. A study identifies EGCG induces cancer cell death in HCC along with evident by promoting production of intracellular ROS upstream of LMP and cell death (Zhang et al., 2012). Then, hydrolytic enzymes (such as cathepsin) in lysosomes leak into the cytoplasm, which in turn triggers non-caspase-dependent cell death (Halaby, 2021).

(-)-Oleocanthal (oleocanthal), a phenolic compound focusing in extra virgin olive oil oleocanthal, has selective toxicity towards HCC cells and reduces cancer incidence by damaging cancer cells’ lysosomes and leak CTSB and CTSD to the cytoplasm *in vitro* and *in vivo* (Goren et al., 2019). The mechanism is targeting the enlarged lysosomes in liver cancer cells and inhibiting the acidic sphingomyelinase ASM, thereby triggering selective LMP and LDCD, without exhibiting significant toxicity to normal liver cells (LeGendre et al., 2015), (-)-Oleocanthal also increases ROS, leading to lysosomal membrane instability. Moreover, low density lipoproteins reconstituted with DHA (LDL-DHA) even has selective toxicity to liver cancer cells rather than normal hepatocytes (Halaby, 2021).

### 4.3 Flavonoids

Flavonoids are important resources for the discovery of new hepatoprotective and antitumor agents (Wang S. et al., 2023). Flavonoids are comprehensive in functionality and more focus on cell death regulation (e.g., apoptosis, ferroptosis).

For example, quercetin, a wide-spread flavonoid, induces LDCD through ROS-mediated TFEB activation and lysosomal iron mobilization. Quercetin generates ROS that promote TFEB nuclear translocation, leading to lysosomal biogenesis and iron accumulation (Tuli et al., 2023). Lysosomal iron release results in Fenton reactions, lipid peroxidation, and ferroptosis (Al Amin et al., 2025). Moreover, quercetin induces LMP by increasing lysosomal pH and disrupting membrane-associated proteins, thereby activating cathepsin-dependent apoptosis and non-apoptotic death pathways (Gemingnuer et al., 2025).

Nobiletin (5, 6, 7, 8, 3′4′-hexamethoxyflavone) is a major anticancer component extracted from zhishi (Rutaceae) juice. Nobiletin is demonstrated to inhibit the growth of tumor through downregulating expressions of Bcl-2 and COX-2, upregulating expressions of Bax and caspase-3 and decreasing the ratios of Bcl-2/Bax *in vitro* and *in vivo* (Ma et al., 2014). After conformational changes, Bax can realize the position transfer from the cytoplasm to the lysosomal membrane and play an inducing role in lysosomal membrane permeability (Kågedal et al., 2005).

Flavonoid compound V8 binds to lysosomal HSP70 through hydrogen bonds, disrupting its interaction with BMP, and leading to pathological sphingomyelin accumulation. SM overload allosterism inhibits TRPML 1, blocks activation of calcium neurotin PPP3CB and subsequent TFEB dephosphorylation. Increased sphingomyelin sabotages lysosomal resilience, with enhanced lysophagy and failed TFEB-driven biogenesis, lysosomal is catastrophic bankrupt (Chen et al., 2025).

Resveratrol, a stilbene found in grapes and berries, targets v-ATPase to inhibit lysosomal acidification, which in turn reduces the stability of tumor lysosomal membrane and promotes rupture (Gemingnuer et al., 2025). Moreover, flavones (Chrysin, luteolin and apigenin) and flavanones (hesperetin and DMHP) have been confirmed that they can decrease the amount of Bcl-2, increase the amount of Bax and reduce the Bcl-2/Bax ratio (He et al., 2019). In addition, apigenin inhibits the PI3K/AKT signaling pathway and increases ROS to trigger apoptosis and autophagy in HCC. Apigenin’s construction enables itself to have toxicity and selectivity towards cancer cells (Yang et al., 2023). Furthermore, baicalin, a Scutellaria baicalensis-extracted flavonoid glycoside, can elevate lysosomal pH, leading to the death of non-small cell lung cancer cells. Baicalin may have potential to be proved the same effect in HCC (Dong X. et al., 2024). Besides, Icariside II, a natural plant flavonoid, upregulates the expression of cytosolic LAMP-1, inducing lysosomal membrane damage and reducing the survival rate of HCC cells (Halaby, 2021).

### 4.4 Artemisinin derivatives

Artemisinin derivatives retain the artemisinin parent nucleus, 1,2,4-trioxane (peroxybridge), have stronger activity and expands new anti-tumor activity.

Artesunate (ART) and dihydroartemisinin (DHA) are semisynthetic derivatives of the antimalarial artemisinin. In HCC cells, ART selectively accumulates in lysosomes and activates cathepsin B and L (CTSB/CTSL), promoting ferritinophagy-mediated iron release and lipid peroxidation by impairment of SLC7A11 (the upstream cystine transporter, causing cell sensitiveness to lipid peroxidation and ferroptosis) (Li et al., 2021). The released Fe^2+^ triggers Fenton chemistry, generating ROS that propagate lipid peroxidation across cellular membranes and inactivate GPX4 (Rodriguez et al., 2024). Further, oxidative damage is accelerated, ultimately leading to LDCD. Another artemisinin derivative, DHA, promotes lipid peroxidation by increasing PEBP1 protein expression, thus activating 15-lipoxygenase (PEBP1/15-LO) and induces ferroptosis (Su et al., 2021).

### 4.5 Esters

Bifendate (DDB), a synthetic intermediate of Schisandrin C extracted from Schisandrae chinensis, is a compound used to treat viral hepatitis and drug-induced liver injury and has anti-HBV properties. DDB exposure significantly increases the LC3-II and p62 expression in ATG5 WT MEFs, suggesting that DDB inhibits canonical autophagy and autophagic degradation by targeting ATG5 and then impairs lysosomal function along with autophagic lysosome reformation. DBB also inhibits autophagosome-lysosome fusion by decreasing SNAP29-STX17-VAMP8 SNARE complex formation, which is in charge of autophagosome-lysosome fusion (Yuan et al., 2022).

Ent-13-Hydroxy-15-kaurene-19-acid N-Methylpiperazine Ethyl Ester (StN), a novel derivative of the natural diterpene stevioside isolated from Stevia rebaudiana (Bertoni), disrupts lysosomal stability and promotes the release of cathepsin B from lysosomes into the nucleus, thereby inducing DNA damage (Guo et al., 2024).

Arenobufagin, a natural bufadienolide from toad venom, inhibits the PI3K/AKT/mTOR pathway to stimulate apoptosis and autophagy in HCC cells (Liu B. et al., 2024).

### 4.6 Alkaloid

Alkaloids with obvious biological activity in natural products are important effective ingredients of the medicinal activity or toxicity of plants. Studies have shown that the alkaloids have antitumor activities. With potential medicinal activity, alkaloids are significant sources of compounds in progressive drug development (Duan et al., 2022).

Chen found that nitidine chloride (NC), a natural phytochemical alkaloid derived from Zanthoxylum nitidum (Roxb.) DC, inhibits mTORC1 activity by targeting amino acid sensing signals and induced activation of transcription factor 4 (ATF4)-mediated upregulation of Sestrin2 expression. NC also binds directly to the insulin-like growth factor 2 receptor (IGF2R) and promotes its lysosome-dependent degradation (Chen F. et al., 2024).

Daurisoline (DAS), as a constituent of Rhizoma Menispermi, is a potential autophagy inhibitor. DAS upregulates autophagosomes along with the increases of LC3-II and p62, demonstrating the influence of autophagic flux. Instead of suppressing the fusion of autophagosomes and lysosomes, DAS reduces lysosomal acidification. Subsequently, mature cathepsin B and cathepsin D decrease in DAS-treated HCC cells. It also promoted the effectiveness of cisplatin (cDDP) for HCC (Xue and Liu, 2021).

Echium amoenum is an annual herb native to the northern mountains of Iran. Research shows E. amoenum’s toxic effects on hepG2 cells probably through lysosomal damage induced by oxidative stress (Zarei et al., 2023).

### 4.7 Others

Chloroquine (CQ) can accumulate in lysosome and prevent endosomal acidification. CQ is an ASM modulator, which displaces ASM from lysosome’s vesicular membranes to reduce ASM activity. ASM is mainly located within the lysosome, and ASM activity is lower in HCC cells leading to higher sphingomyelin levels. HCC cells are susceptible to ASM. Thus, further inhibiting ASM activity can lead to higher levels of sphingomyelin, causing LMP. Therefore, ASM inhibitors like clorpromazine and amiodarone can elicit LMP (Piao and Amaravadi, 2016).

Diallyl sulfide (DAS), as a major component of garlic extracts, inhibits autophagy flow by increasing phosphorylated AMPK and decreasing phosphorylated mTOR to accumulate autophagosomes. Further, DAS raises lysosomal pH, decreases the expression of LAMP1 and blocks cathepsin D maturation, showing the capability of disrupting the integrity of lysosomes, thus blocking autophagic flux (Yu H. et al., 2023).

Zn (II) chelate significantly elevated ROS levels, and specifically localized to lysosomes, leading to lysosomal dysfunction, which is identified through confocal laser scanning microscopy (Mousa et al., 2025).

Moreove, the natural product auraptene, isolated from Citrus aurantium and Aegle marmelos (Tayarani-Najaran et al., 2021), induces total ROS and lipid ROS production in HCC cells leading to ferroptosis and LMP. Besides, auraptene can exert ferroptosis induction by targeting SLC7A11 for ubiquitin–proteasomal degradation (Li et al., 2024a).

Rotenone (Rot), derived from the roots and stems of Derris, Tephrosia, Lonchocarpus and Mundulea plant species (Radad et al., 2019), can elevate mitochondrial reactive oxygen species (mtROS) levels, leading to deteriorated LMP and lysosomal pH elevation. Three lysosomal proteases (m-CTSB, m-CTSD, m-CTSL) increase in the cytoplasm and decrease in lysosomal, and LAMP1 decreases in ROT treatment. Therefore, Rot has potential ability to induce LDCD in HCC cells (Wei et al., 2025).

The study by Wang J. et al. (2024) demonstrates that rhein, derived from the dried root of the Polygonum family palm-leaved rhubarb, directly binds to and inhibits the HSP70 family, thereby acting as a novel HSP72/HSC70/GRP78 inhibitor. Furthermore, the combination of rhein and artemisinin derivatives represents a new approach to treating liver cancer, exhibiting significant antitumor activity both *in vivo* and *in vitro*.

## 5 Limitations and future prospect

The therapeutic potential of natural product targeted at LDCD provides a new direction for the treatment of HCC. However, clinical implementation of LDCD is subject to multiple critical challenges such as selective toxicity, developing personalized treatment plans, drug delivery optimization, and trial design complexity.

### 5.1 Selective toxicity

The selective toxicity of LDCD induced by natural product in HCC is based on fundamental biological differences between malignant and normal hepatocytes. Cancer cells express a hypertrophic lysosome phenotype manifested by increased organelle size, low pH, high protease activity, increased iron content, and alterations in membrane lipid composition.

For example, hypertrophic lysosomes exist in HCC cells with highly acidic and high expression of SMPD1. PD and (-)-Oleocanthal are lysosomotropic saponins, which are preferentially enriched in acidic lysosomes, while normal liver cells with standard lysosome morphology are relatively resistant (Wang Y. et al., 2023). Compared with normal cells, cancer cells have higher levels of cholesterol and lipid rafts, dependent on SREBP. PD can increase SREBP-associated proteins and cause saponins and membrane cholesterol accumulation (Chen et al., 2023), leading to changes in cell membrane permeability, lipid raft destruction, and mitochondrial membrane damage (Lorent et al., 2014). In contrast, normal hepatocytes have a strong homeostasis of lipid metabolism and are not easily disturbed. Saponin extraction from Alcea rosea L. shows high selectivity to Huh-7 cells (SI = 6.62) by contrast with normal HUVEC cells (Abutaha et al., 2024). Besides, the development of liver-targeted formulations can reduce the toxic effects of high dose of NPs and improve efficacy (Wang et al., 2022).

To solve the poor selectivity of curcumin, Wang’s team used a dual-targeted lipid material with low toxicity contained galactose group (recognizing overexpressed ASGPR on HCC) and morpholine group (targeting to the lysosome). This strategy effectively overcomes the off-target distribution problem of curcumin through the dual recognition of “cell surface receptor-suborganelle microenvironment,” and provides a precise delivery platform for lysosomal targeted therapy of liver cancer (Wang Y. et al., 2021).

Zheng et al. (2024) suggest that the use of artificial intelligence can also predict the cytotoxic effects of natural medicines on liver cancer cells and their toxicity to healthy liver tissue. For example, it can optimize the development of polyphenolic drugs to address the current issues of low bioavailability and inconsistent efficacy in liver cancer treatment. This approach could lead to the design of safer and more effective anti-liver cancer drugs (Zheng et al., 2024).

These mechanisms collectively indicate that LDCD therapy based on natural products can exploit the fatal weakness of lysosomes in malignant tumors without damaging liver function, thereby effectively addressing the key issue of hepatotoxicity faced by traditional liver cancer treatment methods.

### 5.2 Drugability

The clinical translation of natural products is limited by their poor water solubility, chemical instability, rapid metabolism, and limited bioavailability (Yu et al., 2026). Most drugs derived from natural products are either highly soluble and poorly metabolized, or highly soluble and rapidly metabolized; however, there are still some drugs that, despite their high bioactivity, are both poorly soluble and poorly metabolized (Li et al., 2016). For example, curcumin has potential as an anti-HCC agent and can modulate multiple signaling pathways (such as the induced production of ROS and AMPK-JNK pathway). However, curcumin has extremely low bioavailability and is highly hydrophobic. Its activity depends on the specific molecular environment and delivery method (Muteeb et al., 2025). When administered orally, the concentration of free drug at the tumor site is unlikely to reach the threshold required to effectively induce cell death. Furthermore, a common limitation of *in vitro* studies on complex natural extracts is the failure to account for the quantitative relationship between free drug concentration and efficacy. Efficacy is judged solely based on the total dose administered, which may lead to false-positive conclusions (Esmaeli et al., 2025).

### 5.3 Drug delivery

The clinical application of natural products is often limited by poor bioavailability, rapid metabolism and off-target distribution. We need appropriate delivery methods to solve these problems.

A study fabricated carrier-free self-assembled nanoparticles CE-Gal-NPs via the nanoprecipitation method. It can actively target ASGPR receptors through galactose ligands, and at the same time use the EPR effect to passively enrich in tumor tissues, enter cells through the clathrin-mediated endocytotic pathway, and finally focus on lysosomes (Zhang X. et al., 2024).

Nanodelivery systems offer a transformative solution to overcome these limitations. However, significant barriers to clinical translation persist. The efficiency of nanoparticle vesicles escaping from their cellular compartments is extremely low (only 1%–2% in hepatocytes), with the vast majority of internalized materials either degraded within lysosomes or recycled through exocytosis (Rosenblum et al., 2018). Additionally, the complex chemical structures involved in stimulus-response nanocarriers further complicate drug development, scalability, and regulatory approvals. Simplified designs based on exogenous stimuli (temperature, magnetic field) have entered clinical trials (e.g., ThermoDox), while endogenous stimulus response systems (pH, enzymes) are challenged due to differences between preclinical models and human tumors.

The development of intelligent nanocarriers that integrate lysosomal targeting, enhanced optical properties and microenvironment-responsive drug release, such as Zn(II)-Schiff base complexes for lysosomal theranostics, represents a promising direction for diagnostic precision and therapeutic efficacy (Lai et al., 2025).

### 5.4 Safety

Although natural products that induce LMP can promote cancer cell death, they may exacerbate conditions such as hepatitis and liver fibrosis due to the action of cathepsins (Yang H. et al., 2022).

Current research on natural products inducing apoptosis in HCC primarily focuses on pharmacological effects, such as the ability of natural products to target specific pathways or molecules, or simply examines their impact on cancer-associated inflammation. There is a lack of studies investigating the complete chain of events leading from natural products to the induction of LDCD and harmful inflammation. This may be because these two effects appear to be distinct. However, LDCD is a complex network of mechanisms that interacts with other pathways, and exploring the connections between them may represent a promising direction for HCC treatment.

The goal of using natural products to induce LDCD is to achieve localized delivery. However, the systemic risks associated with off-target drug delivery during the delivery process should not be overlooked.

When an increase in lysosomal pH and impaired lysosomal function occur in various cells throughout the body, they are associated with the onset and progression of various diseases. Study has shown that impairment of lysosomal acidification significantly increases the body’s susceptibility to pathogens. This implies that anticancer strategies that disrupt lysosomal acidification may expose patients to a higher risk of infection (Goering et al., 2025).

Researchers are working to use nanotechnology to enhance the bioactivity of natural products and achieve targeted accumulation at the site of the lesion. However, nanotechnology also poses certain safety concerns. Lipid-based nanocarriers have limited drug-loading capacity and are prone to lipid oxidation. Inorganic nanocarriers may persist in the body for extended periods if their particle size or surface modifications prevent degradation, potentially triggering sustained inflammatory responses or organ damage. The degradation of polymeric nanoparticles may produce acidic byproducts, which can cause tissue irritation if local concentrations become too high. Furthermore, while surface-modified polyethylene glycol (PEG) can prolong circulation time, repeated administration may induce the body to produce anti-PEG antibodies, triggering an immune response (Yu et al., 2026). Most current studies focus on short-term efficacy and acute toxicity. There is a lack of sufficient safety data to address issues related to the source of toxicity, accumulation, or genetic effects.

### 5.5 Clinical challenges

Preclinical research models for HCC typically utilize 2D cell lines (such as HepG2, Huh7, and Hep3B), organoids, xenograft models, patient-derived xenografts (PDX), genetically engineered mouse (GEM) models, and hydrodynamic transfection (HTVI) models. Gu et al. (2015) demonstrates that the histological characteristics and gene expression patterns of PDX models are highly consistent with those of xenograft tumors and their corresponding primary tumors. PDX models are regarded as clinically relevant platforms for drug screening, biomarker discovery, and translational research in a preclinical setting. Traditional xenograft models (including 2D cell xenografts and PDX) typically require the use of immunodeficient mice to prevent rejection. This indicates that these models lack a functional human immune system. Human HCC typically develops against a background of chronic hepatitis or cirrhosis. Although preclinical models can simulate some of these conditions, it is difficult to fully replicate the complex chronic inflammatory and fibrotic processes observed in humans within animal models. Furthermore, in subcutaneous tumor models, tumor growth occurs under the skin rather than in the spleen, leading to differences in the microenvironment (Cigliano et al., 2024).

Genetically Engineered Model Mice (GEMMs) can replicate the entire course of disease progression and support long-term assessment of biomarkers and immunometabolism. In this regard, they are superior to cell line and organoid models (Liapopoulos et al., 2025).

Given the characteristics of these models, conducting preclinical studies to investigate the therapeutic potential of natural products for HCC in preparation for clinical translation requires the use of multiple models.

### 5.6 Individualized design

The key to the clinical translation of LDCD targeting natural products is to identify predictive biomarkers to stratify patient populations. SMPD1 expression levels and lysosomal sphingomyelin content are promising candidate biomarkers, as evidenced by the selective efficacy of polyphyllin D in SMPD1-high HCC cells (Wu Y. P. et al., 2025). Similarly, LAMP-1 and LAMP-2 expression level or LysoTracker uptake intensity in tumor tissue could identify patients most likely to respond to LMP-inducing agents (Zarei et al., 2023). However, there is a lack of standardized detection methods for these biomarkers in current clinical practice, and their prognostic predictive value in patients with hepatocellular carcinoma of different etiologies (viral hepatitis, alcohol, NASH) has not been clearly demonstrated.

In the treatment of LDCD, the precise selection of lysosomal membrane permeabilization (LMP) or lysosomal membrane rupture (LMR) pathways is key to achieving therapeutic goals. Although both mechanisms lead to cathepsin release, there are essential differences in the nature of membrane damage, cell death phenotype, and biological consequences. LMP manifests as leakage through the ultrastructurally-intact limiting membrane, mediating selective and progressive content release, and ultimately activating caspase-dependent apoptosis programs. LMR, on the other hand, is characterized by an interruption of membrane continuity visible by electron microscopy, resulting in acute, large-scale hydrolase leakage, triggering necrotic cell death. Therefore, the selection of treatment strategies needs to be comprehensively considered based on the characteristics of the tumor microenvironment and treatment goals. For acute, intense stress conditions (like ischemia, heat shock, radiation, or irreversible oxidative stress), the induction of the LMR pathway is preferred. LMR performs rapid cell disintegration by calpain-cathepsin cascade. This process can be achieved with lipid peroxidation products such as 4-Hydroxy-2-nonenal (4-HNE) or specific nanomaterials. Conversely, LMP-mediated apoptosis pathways are more appropriate for mild or chronic stress conditions (like oxidative stress, growth factor deprivation, endoplasmic reticulum stress) because they allow cells to die programmed, and lysosomal structure is maintained until death is complete (Yamashima, 2025).

In summary, the optimization of LDCD treatment strategies requires the integration of stress intensity, cell death resistance mechanism, and membrane markers functional status for individualized design. The best treatment effect is achieved by precisely regulating lysosomal membrane damage.

### 5.7 Combination strategies

The “prime-and-kill” strategy, combining TFEB activators (e.g., curcumin, resveratrol) with direct LMP inducers (e.g., polyphyllin D, artesunate), is expected to improve treatment indices by preloading autophagy substrates that amplifies the lethality of subsequent membrane permeabilization.

It is identified that the combination of sodium butyrate (NaB, autophagy inducer) and chloroquine (CQ, autophagy blocker) produced a significant synergistic killing effect on all human hepatocytic carcinoma cell lines and was non-toxic to normal hepatocytes. This combined tactic leads to the death of cancer cells by inducing autophagosome accumulation, increased lysosomal membrane permeability and elevated oxidative stress (Shalhoub et al., 2024). In addition, the combination of the flavonoid Isoliquiritigenin (ISL) from licorice root and arsenic trioxide (ATO) was found to exhibit significant anti-hepatocellular cancer effects both *in vitro* and *in vivo*. This effect is achieved by synergistically inducing mitochondrial apoptosis (dependent on the production of ROS) and inhibiting the PI3K/Akt/mTOR signaling pathway (Li J. et al., 2025). The results suggest that simultaneous activation and blocking of autophagy may be an effective strategy against liver cancer.

Moreover, agrimol B, a natural polyphenol, significantly enhances the sensitivity of HCC cells to sorafenib *in vitro* and *in vivo* (Dong L. et al., 2024). The combination of ART with low-dose sorafenib can synergistically induce ferroptosis in HCC cell lines and nude mouse xenografts, thereby overcoming the common problem of apoptosis resistance in monotherapy (Li et al., 2021). Astragali radix - curcumae rhizoma herb pair (ACHP) enhances the efficacy of sorafenib by inducing lipid peroxidation-related ferroptosis (Wang C. et al., 2025). Manoalide is a potential EGFR-TKI sensitizer for lung cancer cells harboring KRAS mutations and resistant to osimertinib. By inhibiting the ROS-mediated KRAS-ERK pathway and inducing ferroptosis through mitochondrial calcium overload, it effectively overcomes EGFR-TKI resistance in lung cancer (Ni et al., 2022). Sertraline and fluoxetine are selective serotonin reuptake inhibitors (SSRIs). Sertraline and fluoxetine can synergize with sorafenib by inhibiting AKT/mTOR pathway and suppress the viability of HCC (Zhang H. et al., 2021). These suggest that some natural products can enhance the efficacy of traditional drugs for the treatment of liver cancer and reduce drug resistance, or they can combine with other mechanisms to fight cancer.

## 6 Conclusion

The concept of “lysosomal addiction,” the unique metabolic dependency cancer cells on lysosomal function for survival, growth, Targeting lysosome may provide exploitable therapeutic windows for future precision medicine approaches development in HCC, either by inducing cell death or by promoting the release of therapeutic drug targeting endocytic pathways (Alu et al., 2020; Li et al., 2024b). And natural product-induced lysosome-dependent cell death represents a paradigm shift in liver cancer treatment, transforming malignant lysosomes from pro-survival adaptations to lethal fragility. The diversity of chemical structures (like sesquiterpenes, saponins, alkaloids, flavonoids) and mechanisms (like SMPD1 inhibition, iron mobilization, pH modulation, oxidative Stress, TFEB activation) provides robust tools for addressing the heterogeneity and drug resistance that limit current treatments in HCC.

## References

[B1] Abutaha N. M. T. Alghamdi R. Al-Waddan M. (2024). Induction of apoptosis and ROS production in liver cancer cells by saponin fraction from Alcea rosea L. seeds. Indian J. Animal Res. 58 (10), 1765–1771. 10.18805/ijar.bf-1809

[B2] Aits S. Jäättelä M. (2013). Lysosomal cell death at a glance. J. Cell Sci. 126, 1905–1912. 10.1242/jcs.091181 23720375

[B3] Al Amin M. Zehravi M. Sweilam S. H. Jahnavi P. Gupta J. K. Rajashakar V. (2025). Natural agents modulating ferroptosis in cancer: molecular pathways and therapeutic perspectives. J. Cell Mol. Med. 29, e70834. 10.1111/jcmm.70834 40916076 PMC12414808

[B4] Alu A. Han X. Ma X. Wu M. Wei Y. Wei X. (2020). The role of lysosome in regulated necrosis. Acta Pharm. Sin. B 10, 1880–1903. 10.1016/j.apsb.2020.07.003 33163342 PMC7606114

[B5] An J. Zhang L. Duan Y. Pu S. Peng F. (2026). Sodium's role and therapeutic targeting in cancer. Trends Pharmacol. Sci. 47, 53–65. 10.1016/j.tips.2025.10.015 41318248

[B6] Berg A. L. Rowson-Hodel A. Wheeler M. R. Hu M. Free S. R. Carraway K. L. III (2022). “Engaging the lysosome and lysosome-dependent cell death in cancer,” in Breast Cancer. Editor Mayrovitz H. N. (Brisbane, AU: Exon Publications). 36122163

[B7] Bi J. Sun Y. Guo M. Sun X. Sun J. Jiang R. (2025). Lysosomes: guardians and healers within cells-multifaceted perspective and outlook from injury repair to disease treatment. Cancer Cell Int. 25, 136. 10.1186/s12935-025-03771-5 40205430 PMC11984033

[B8] Bray F. Laversanne M. Sung H. Ferlay J. Siegel R. L. Soerjomataram I. (2024). Global cancer statistics 2022: GLOBOCAN estimates of incidence and mortality worldwide for 36 cancers in 185 countries. CA: Cancer J. Clin. 74, 229–263. 10.3322/caac.21834 38572751

[B9] Chatzikalil E. Arvanitakis K. Kalopitas G. Florentin M. Germanidis G. Koufakis T. (2025). Hepatic iron overload and hepatocellular carcinoma: new insights into pathophysiological mechanisms and therapeutic approaches. Cancers (Basel) 17, 392. 10.3390/cancers17030392 39941760 PMC11815926

[B10] Chen C. Zhang C. Jin Z. Wu B. Xu T. (2022). Sex differences in immune-related adverse events with immune checkpoint inhibitors: data mining of the FDA adverse event reporting system. Int. J. Clin. Pharm. 44, 689–697. 10.1007/s11096-022-01395-7 35449347

[B11] Chen Y. Y. Ren C. F. Wen S. Y. (2023). Polyphyllin D induces G2/M cell cycle arrest via dysfunction of cholesterol biosynthesis in liver cancer cells. Biomed. Environ. Sci. 36, 94–98. 10.3967/bes2023.009 36650685

[B12] Chen F. Peng S. Li C. Yang F. Yi Y. Chen X. (2024). Nitidine chloride inhibits mTORC1 signaling through ATF4-mediated Sestrin2 induction and targets IGF2R for lysosomal degradation. Life Sci. 353, 122918. 10.1016/j.lfs.2024.122918 39034027

[B13] Chen L. Hu Y. Li Y. Zhang B. Wang J. Deng M. (2024). Integrated multiomics analysis identified comprehensive crosstalk between diverse programmed cell death patterns and novel molecular subtypes in hepatocellular carcinoma. Sci. Rep. 14, 27529. 10.1038/s41598-024-78911-4 39528670 PMC11555373

[B14] Chen H. Zhu M. Zhao Z. Jiang Y. Wang H. Liu Y. (2025). Overcoming ELDR-mediated cancer cell survival *in vitro* and *in vivo* via sphingomyelin-driven TRPML1 inactivation and TFEB suppression through lysosomal HSP70 targeting. Pharmacol. Res. 222, 108050. 10.1016/j.phrs.2025.108050 41352406

[B15] Chevriaux A. Pilot T. Derangère V. Simonin H. Martine P. Chalmin F. (2020). Cathepsin B is required for NLRP3 inflammasome activation in macrophages, through NLRP3 interaction. Front. Cell Dev. Biol. 8, 167. 10.3389/fcell.2020.00167 32328491 PMC7162607

[B16] Cigliano A. Liao W. Deiana G. A. Rizzo D. Chen X. Calvisi D. F. (2024). Preclinical models of hepatocellular carcinoma: current utility, limitations, and challenges. Biomedicines 12, 1624. 10.3390/biomedicines12071624 39062197 PMC11274649

[B17] Courtois A. Marié C. Fouquet G. Diouf M. Esparteiro D. Ducournau G. (2026). Alcohol induces sorafenib resistance in hepatocellular carcinoma: a translational study. J. Mol. Med. (Berl) 104, 34. 10.1007/s00109-026-02645-1 41580527 PMC12831666

[B18] Dai B. Cao H. Hu Y. Gong Z. Huang X. Chen Y. (2023). Role of NLRP3 inflammasome activation in HCC cell progression. Heliyon 9, e19542. 10.1016/j.heliyon.2023.e19542 37681160 PMC10481302

[B19] Deshpande R. O’reilly D. Sherlock D. (2011). Improving outcomes with surgical resection and other ablative therapies in HCC. Int. J. Hepatol. 2011, 686074. 10.4061/2011/686074 21994867 PMC3170839

[B20] Desideri E. Ciriolo M. R. (2021). Inhibition of JNK increases the sensitivity of hepatocellular carcinoma cells to lysosomotropic drugs *via* LAMP2A destabilization. Cell Death Discov. 7, 29. 10.1038/s41420-021-00408-0 33558496 PMC7870977

[B21] Domagala A. Fidyt K. Bobrowicz M. Stachura J. Szczygiel K. Firczuk M. (2018). Typical and atypical inducers of lysosomal cell death: a promising anticancer strategy. Int. J. Mol. Sci. 19 (8), 2256. 10.3390/ijms19082256 30071644 PMC6121368

[B22] Dong H. Guo W. Zhou Z. (2024). BAX/MLKL signaling contributes to lipotoxicity-induced lysosomal membrane permeabilization in alcohol-associated liver disease. Autophagy 20, 958–959. 10.1080/15548627.2023.2221989 37289043 PMC11062378

[B23] Dong L. Luo L. Wang Z. Lian S. Wang M. Wu X. (2024). Targeted degradation of NDUFS1 by agrimol B promotes mitochondrial ROS accumulation and cytotoxic autophagy arrest in hepatocellular carcinoma. Free Radic. Biol. Med. 220, 111–124. 10.1016/j.freeradbiomed.2024.04.242 38697493

[B24] Dong X. Liu X. Lin D. Zhang L. Wu Y. Chang Y. (2024). Baicalin induces cell death of non-small cell lung cancer cells via MCOLN3-mediated lysosomal dysfunction and autophagy blockage. Phytomedicine 133, 155872. 10.1016/j.phymed.2024.155872 39096542

[B25] Duan H. Er-Bu A. Dongzhi Z. Xie H. Ye B. He J. (2022). Alkaloids from Dendrobium and their biosynthetic pathway, biological activity and total synthesis. Phytomedicine 102, 154132. 10.1016/j.phymed.2022.154132 35576743

[B26] Duan J. Huang Z. Qin S. Li B. Zhang Z. Liu R. (2024). Oxidative stress induces extracellular vesicle release by upregulation of HEXB to facilitate tumour growth in experimental hepatocellular carcinoma. J. Extracell. Vesicles 13, e12468. 10.1002/jev2.12468 38944674 PMC11214608

[B27] Esmaeli M. Dehabadi M. D. Ghanbari A. (2025). Molecular targets and therapeutic implications of curcumin in hepatocellular carcinoma: a comprehensive literature review. Cancer Cell Int. 25, 335. 10.1186/s12935-025-03988-4 41044771 PMC12495814

[B28] Ferret L. Pol J. G. Sauvat A. Stoll G. Alvarez-Valadez K. Muller A. (2025). Lysosomal membrane permeabilization enhances the anticancer effects of POLR1 (RNA polymerase I) transcription inhibitors. Autophagy 21, 2246–2265. 10.1080/15548627.2025.2497614 40528705 PMC12582078

[B29] Gemingnuer A. Yin R. Liu Y. Tian Y. Meng X. (2025). Lysosome-dependent cell death: disease implications and potential therapeutic targets. Mol. Biol. Rep. 52, 881. 10.1007/s11033-025-10997-z 40928622

[B30] Geng Y. D. Zhang C. Lei J. L. Yu P. Xia Y. Z. Zhang H. (2017). Walsuronoid B induces mitochondrial and lysosomal dysfunction leading to apoptotic rather than autophagic cell death via ROS/p53 signaling pathways in liver cancer. Biochem. Pharmacol. 142, 71–86. 10.1016/j.bcp.2017.06.134 28673807

[B31] Goering E. R. Clatworthy A. E. Parada-Kusz M. Bagnall J. Hung D. T. (2025). Kmo restricts Salmonella in a whole organism infection model by promoting macrophage lysosomal acidification through kainate receptor antagonism. PLoS Pathog. 21, e1013273. 10.1371/journal.ppat.1013273 41129589 PMC12571273

[B32] Goren L. Zhang G. Kaushik S. Breslin P. a.S. Du Y. N. Foster D. A. (2019). (-)-Oleocanthal and (-)-oleocanthal-rich olive oils induce lysosomal membrane permeabilization in cancer cells. PLoS One 14, e0216024. 10.1371/journal.pone.0216024 31412041 PMC6693737

[B33] Goyal H. Parwani S. Kaur J. (2024). Deciphering the nexus between long non-coding RNAs and endoplasmic reticulum stress in hepatocellular carcinoma: biomarker discovery and therapeutic horizons. Cell Death Discov. 10, 451. 10.1038/s41420-024-02200-2 39448589 PMC11502918

[B34] Gu Q. Zhang B. Sun H. Xu Q. Tan Y. Wang G. (2015). Genomic characterization of a large panel of patient-derived hepatocellular carcinoma xenograft tumor models for preclinical development. Oncotarget 6, 20160–20176. 10.18632/oncotarget.3969 26062443 PMC4652995

[B35] Guo Y. Wang S. Dong Y. Liu Y. (2024). Attenuation of pro-tumorigenic senescent secretory phenotype by StN, a novel derivative of stevioside, potentiates its inhibitory activity on hepatocellular carcinoma. Food Chem. Toxicol. 184, 114371. 10.1016/j.fct.2023.114371 38104710

[B36] Halaby R. (2021). Natural products induce lysosomal membrane permeabilization as an anticancer strategy. Med. (Basel) 8, 69. 10.3390/medicines8110069 PMC8624533 34822366

[B37] He Y. Xia Z. Yu D. Wang J. Jin L. Huang D. (2019). Hepatoprotective effects and structure-activity relationship of five flavonoids against lipopolysaccharide/d-galactosamine induced acute liver failure in mice. Int. Immunopharmacol. 68, 171–178. 10.1016/j.intimp.2018.12.059 30641432

[B38] He S. Sun J. Guan H. Su J. Chen X. Hong Z. (2024). Molecular characteristics and prognostic significances of lysosomal-dependent cell death in kidney renal clear cell carcinoma. Aging (Albany NY) 16, 4862–4888. 10.18632/aging.205639 38460947 PMC10968703

[B39] Ho J. K. H. Thurairajah P. H. Leo J. Huang D. Q. Y. Fan K. H. (2024). Sex differences in hepatocellular carcinoma. Hepatoma Res. 10, 53. 10.20517/2394-5079.2024.119

[B40] Hong J. M. Kim J. H. Kim H. Lee W. J. Hwang Y. I. (2019). SB365, Pulsatilla saponin D induces caspase-independent cell death and augments the anticancer effect of temozolomide in glioblastoma multiforme cells. Molecules 24 (18), 3230. 10.3390/molecules24183230 31491945 PMC6766801

[B41] Hwang S. Y. Danpanichkul P. Agopian V. Mehta N. Parikh N. D. Abou-Alfa G. K. (2025). Hepatocellular carcinoma: updates on epidemiology, surveillance, diagnosis and treatment. Clin. Mol. Hepatol. 31, S228–s254. 10.3350/cmh.2024.0824 39722614 PMC11925437

[B42] Jeon Y. Kwon H. Chung T. Park Y. N. Kim S. N. Park J. Y. (2025). Notoginsenoside Ft1 induces lysosomal cell death and apoptosis by inhibiting the PI3K/AKT/mTOR pathway in hepatocellular carcinoma. Biomed. Pharmacother. 188, 118181. 10.1016/j.biopha.2025.118181 40451034

[B43] Jin Y. Wang C. Meng Z. Zhang Y. Meng D. Liu J. (2025). Proanthocyanidins alleviate acute alcohol liver injury by inhibiting pyroptosis via inhibiting the ROS-MLKL-CTSB-NLRP3 pathway. Phytomedicine 136, 156268. 10.1016/j.phymed.2024.156268 39612889

[B44] Joerg V. Scheiner B. A D. A. Fulgenzi C. a.M. Schönlein M. Kocheise L. (2023). Efficacy and safety of atezolizumab/bevacizumab in patients with HCC after prior systemic therapy: a global, observational study. Hepatol. Commun. 7 (11) e0302. 10.1097/HC9.0000000000000302 37889520 PMC10615429

[B45] Kågedal K. Johansson A. C. Johansson U. Heimlich G. Roberg K. Wang N. S. (2005). Lysosomal membrane permeabilization during apoptosis—involvement of Bax? Int. J. Exp. Pathol. 86, 309–321. 10.1111/j.0959-9673.2005.00442.x 16191103 PMC2517437

[B46] Kim J. H. Land K. M. Huang C. Zhang Y. Y. (2023). Natural products as drug candidates for redox-related human disease. Pharm. (Basel) 16, 1294. 10.3390/ph16091294 PMC10536196 37765102

[B47] Kudo M. (2022). Durvalumab plus tremelimumab: a novel combination immunotherapy for unresectable hepatocellular carcinoma. Liver Cancer 11, 87–93. 10.1159/000523702 35634425 PMC9109076

[B48] Lai X. Lu T. Zhang F. Khan A. Zhao Y. Li X. (2025). Lysosome-targeted theranostics: integration of real-time fluorescence imaging and controlled drug delivery via Zn(II)-Schiff base complexes. J. Inorg. Biochem. 272, 113015. 10.1016/j.jinorgbio.2025.113015 40716183

[B49] Legendre O. Breslin P. A. Foster D. A. (2015). (-)-Oleocanthal rapidly and selectively induces cancer cell death via lysosomal membrane permeabilization. Mol. Cell Oncol. 2, e1006077. 10.1080/23723556.2015.1006077 26380379 PMC4568762

[B50] Li F. Fernandez P. P. Rajendran P. Hui K. M. Sethi G. (2010). Diosgenin, a steroidal saponin, inhibits STAT3 signaling pathway leading to suppression of proliferation and chemosensitization of human hepatocellular carcinoma cells. Cancer Lett. 292, 197–207. 10.1016/j.canlet.2009.12.003 20053498

[B51] Li J. Larregieu C. A. Benet L. Z. (2016). Classification of natural products as sources of drugs according to the biopharmaceutics drug disposition classification system (BDDCS). Chin. J. Nat. Med. 14, 888–897. 10.1016/S1875-5364(17)30013-4 28262115

[B52] Li Z. J. Dai H. Q. Huang X. W. Feng J. Deng J. H. Wang Z. X. (2021). Artesunate synergizes with sorafenib to induce ferroptosis in hepatocellular carcinoma. Acta Pharmacol. Sin. 42, 301–310. 10.1038/s41401-020-0478-3 32699265 PMC8026986

[B53] Li D. Li Y. Chen L. Gao C. Dai B. Yu W. (2024a). Natural product auraptene targets SLC7A11 for degradation and induces hepatocellular carcinoma ferroptosis. Antioxidants (Basel) 13, 1015. 10.3390/antiox13081015 39199259 PMC11351406

[B54] Li D. Wang J. Tuo Z. Yoo K. H. Yu Q. Miyamoto A. (2024b). Natural products and derivatives in renal, urothelial and testicular cancers: targeting signaling pathways and therapeutic potential. Phytomedicine 127, 155503. 10.1016/j.phymed.2024.155503 38490077

[B55] Li J. Gu J. Pan S. Deng N. Khan M. Li L. (2025). Synergic effect of the combination of isoliquiritigenin and arsenic trioxide in HepG2 liver cancer cells. Cell Signal 131, 111752. 10.1016/j.cellsig.2025.111752 40107478

[B56] Li L. Xu X. Wang W. Huang P. Yu L. Ren Z. (2025). Safety and efficacy of PD-1 inhibitor (sintilimab) combined with transarterial chemoembolization as the initial treatment in patients with intermediate-stage hepatocellular carcinoma beyond up-to-seven criteria. J. Immunother. Cancer 13, e010035. 10.1136/jitc-2024-010035 39824532 PMC11749212

[B57] Liapopoulos D. Sarantis P. Biniari T. Bousou T. E. Trifylli E. M. Anastasiou I. A. (2025). MicroRNA landscape in hepatocellular carcinoma: Metabolic re-wiring, predictive and diagnostic biomarkers, and emerging therapeutic targets. Biomedicines 13, 2243. 10.3390/biomedicines13092243 41007803 PMC12467513

[B58] Liu S. Yang T. Ming T. W. Gaun T. K. W. Zhou T. Wang S. (2020). Isosteroid alkaloids from Fritillaria cirrhosa bulbus as inhibitors of cigarette smoke-induced oxidative stress. Fitoterapia 140, 104434. 10.1016/j.fitote.2019.104434 31760067

[B59] Liu T. Li Q. Lin Z. Wang P. Chen Y. Fu Y. (2021). Viral infections and the efficacy of PD-(L)1 inhibitors in virus-related cancers: head and neck squamous cell carcinoma and hepatocellular carcinoma. Int. Immunopharmacol. 100, 108128. 10.1016/j.intimp.2021.108128 34537483

[B60] Liu H. Y. Lee Y. D. Sridharan S. Wang W. Khor R. Chu J. (2023). Definitive stereotactic body radiation therapy in early-stage solitary hepatocellular carcinoma: an Australian multi-institutional review of outcomes. Clin. Oncol. (R Coll. Radiol) 35, 787–793. 10.1016/j.clon.2023.08.012 37709623

[B61] Liu B. Liu L. Liu Y. (2024). Targeting cell death mechanisms: the potential of autophagy and ferroptosis in hepatocellular carcinoma therapy. Front. Immunol. 15, 1450487. 10.3389/fimmu.2024.1450487 39315094 PMC11416969

[B62] Liu Y. Wang F. Yan G. Tong Y. Guo W. Li S. (2024). CPT1A loss disrupts BCAA metabolism to confer therapeutic vulnerability in TP53-mutated liver cancer. Cancer Lett. 595, 217006. 10.1016/j.canlet.2024.217006 38823763

[B63] Liu H. Zhang Y. Lv X. Ding X. Liao W. Sun W. (2025). Breaking the bottlenecks in anti-tumor angiogenic therapy: targeting vasculogenic mimicry with natural products and traditional Chinese medicine. Front. Pharmacol. 16, 1668083. 10.3389/fphar.2025.1668083 41019994 PMC12464493

[B64] Long F. Y. Chen Y. S. Zhang L. Kuang X. Yu Y. Wang L. F. (2015). Pennogenyl saponins induce cell cycle arrest and apoptosis in human hepatocellular carcinoma HepG2 cells. J. Ethnopharmacol. 162, 112–120. 10.1016/j.jep.2014.12.065 25562722

[B65] Lorent J. H. Quetin-Leclercq J. Mingeot-Leclercq M. P. (2014). The amphiphilic nature of saponins and their effects on artificial and biological membranes and potential consequences for red blood and cancer cells. Org. Biomol. Chem. 12, 8803–8822. 10.1039/c4ob01652a 25295776

[B66] Ma X. Jin S. Zhang Y. Wan L. Zhao Y. Zhou L. (2014). Inhibitory effects of nobiletin on hepatocellular carcinoma *in vitro* and *in vivo* . Phytother. Res. 28, 560–567. 10.1002/ptr.5024 23818450

[B67] Mauro E. De Castro T. Zeitlhoefler M. Sung M. W. Villanueva A. Mazzaferro V. (2025). Hepatocellular carcinoma: epidemiology, diagnosis and treatment. JHEP Rep. 7, 101571. 10.1016/j.jhepr.2025.101571 41244300 PMC12615749

[B68] Mir I. H. Thirunavukkarasu C. (2023). The relevance of acid sphingomyelinase as a potential target for therapeutic intervention in hepatic disorders: current scenario and anticipated trends. Arch. Toxicol. 97, 2069–2087. 10.1007/s00204-023-03529-w 37248308 PMC10226719

[B69] Mousa I. Ageeli A. A. Siddiq H. A. Alkhathami N. D. Alatawi N. M. Alenazy D. M. (2025). Synthesis, characterization, and anticancer evaluation of nano-sized schiff base metal chelates. Biometals 38, 2043–2073. 10.1007/s10534-025-00746-x 41310122

[B70] Muteeb G. El-Morsy M. T. Abo-Taleb M. A. Mohamed S. K. Khafaga D. S. R. (2025). Herbal medicine: enhancing the anticancer potential of natural products in hepatocellular carcinoma therapy through advanced drug delivery systems. Pharmaceutics 17, 673. 10.3390/pharmaceutics17050673 40430962 PMC12114929

[B71] Ni Y. Liu J. Zeng L. Yang Y. Liu L. Yao M. (2022). Natural product manoalide promotes EGFR-TKI sensitivity of lung cancer cells by KRAS-ERK pathway and mitochondrial Ca(2+) overload-induced ferroptosis. Front. Pharmacol. 13, 1109822. 10.3389/fphar.2022.1109822 36712673 PMC9873971

[B72] Pandey P. Elsori D. Kumar R. Lakhanpal S. Rautela I. Alqahtani T. M. (2024). Ferroptosis targeting natural compounds as a promising approach for developing potent liver cancer agents. Front. Pharmacol. 15, 1399677. 10.3389/fphar.2024.1399677 38738178 PMC11082342

[B73] Pang Z. (2025). Copper metabolism in hepatocellular carcinoma: from molecular mechanisms to therapeutic opportunities. Front. Mol. Biosci. 12, 1578693. 10.3389/fmolb.2025.1578693 40433591 PMC12106024

[B74] Piao S. Amaravadi R. K. (2016). Targeting the lysosome in cancer. Ann. N. Y. Acad. Sci. 1371, 45–54. 10.1111/nyas.12953 26599426 PMC4879098

[B75] Pihán P. Lisbona F. Borgonovo J. Edwards-Jorquera S. Nunes-Hasler P. Castillo K. (2021). Control of lysosomal-mediated cell death by the pH-dependent calcium channel RECS1. Sci. Adv. 7, eabe5469. 10.1126/sciadv.abe5469 34767445 PMC8589314

[B76] Qi D. Qin Y. Zhu H. Li Y. Han S. (2025). Resistance of first-line targeted drugs in hepatocellular carcinoma: the epigenetic regulation mechanisms. Cell Death Dis. 16, 875. 10.1038/s41419-025-08105-x 41298358 PMC12669607

[B77] Radad K. Al-Shraim M. Al-Emam A. Wang F. Kranner B. Rausch W. D. (2019). Rotenone: from modelling to implication in Parkinson's disease. Folia Neuropathol. 57, 317–326. 10.5114/fn.2019.89857 32337944

[B78] Radulovic M. Yang C. Stenmark H. (2026). Lysosomal membrane homeostasis and its importance in physiology and disease. Nat. Rev. Mol. Cell Biol. 27, 71–87. 10.1038/s41580-025-00873-w 40759742

[B79] Rainey N. E. Moustapha A. Petit P. X. (2020). Curcumin, a multifaceted hormetic agent, mediates an intricate crosstalk between mitochondrial turnover, autophagy, and apoptosis. Oxid. Med. Cell Longev. 2020, 3656419. 10.1155/2020/3656419 32765806 PMC7387956

[B80] Ren W. Jiang H. Song Q. Chen Y. Tang C. Wang F. (2025). TCF25 serves as a nutrient sensor to orchestrate metabolic adaptation and cell death by enhancing lysosomal acidification under glucose starvation. Cell Rep. 44, 116186. 10.1016/j.celrep.2025.116186 40844875 PMC13010378

[B81] Rodriguez R. Cañeque T. Baron L. Müller S. Carmona A. Colombeau L. (2024). Activation of lysosomal iron triggers ferroptosis in cancer. Res. Sq. 10.21203/rs.3.rs-4165774/v1 PMC12158755 40335696

[B82] Rosenblum D. Joshi N. Tao W. Karp J. M. Peer D. (2018). Progress and challenges towards targeted delivery of cancer therapeutics. Nat. Commun. 9, 1410. 10.1038/s41467-018-03705-y 29650952 PMC5897557

[B83] Sakamaki J. I. Wilkinson S. Hahn M. Tasdemir N. O'prey J. Clark W. (2017). Bromodomain protein BRD4 is a transcriptional repressor of autophagy and lysosomal function. Mol. Cell 66, 517–532.e519. 10.1016/j.molcel.2017.04.027 28525743 PMC5446411

[B84] Sardiello M. Palmieri M. Di Ronza A. Medina D. L. Valenza M. Gennarino V. A. (2009). A gene network regulating lysosomal biogenesis and function. Science 325, 473–477. 10.1126/science.1174447 19556463

[B85] Sequeira L. M. Ozturk N. B. Sierra L. Gurakar M. Toruner M. D. Zheng M. (2025). Hepatocellular carcinoma and the role of liver transplantation: an update and review. J. Clin. Transl. Hepatol. 13, 327–338. 10.14218/JCTH.2024.00432 40206277 PMC11976436

[B86] Shalhoub H. Gonzalez P. Dos Santos A. Guillermet-Guibert J. Moniaux N. Dupont N. (2024). Simultaneous activation and blockade of autophagy to fight hepatocellular carcinoma. Autophagy Rep. 3, 2326241. 10.1080/27694127.2024.2326241 40395533 PMC11864649

[B87] Shen J. Y. Li C. Wen T. F. Yan L. N. Li B. Wang W. T. (2016). Liver transplantation versus surgical resection for HCC meeting the Milan criteria: a propensity score analysis. Med. (Baltimore) 95, e5756. 10.1097/MD.0000000000005756 28033289 PMC5207585

[B88] Sonbol M. B. Riaz I. B. Naqvi S. a.A. Almquist D. R. Mina S. Almasri J. (2020). Systemic therapy and sequencing options in advanced hepatocellular carcinoma: a systematic review and network meta-analysis. JAMA Oncol. 6, e204930. 10.1001/jamaoncol.2020.4930 33090186 PMC7582230

[B89] Spiegel S. Merrill A. H. Jr. (1996). Sphingolipid metabolism and cell growth regulation. Faseb J. 10, 1388–1397. 10.1096/fasebj.10.12.8903509 8903509

[B90] Su Y. Zhao D. Jin C. Li Z. Sun S. Xia S. (2021). Dihydroartemisinin induces ferroptosis in HCC by promoting the formation of PEBP1/15-LO. Oxid. Med. Cell Longev. 2021, 3456725. 10.1155/2021/3456725 34925691 PMC8683180

[B91] Takuwa Y. Okamoto Y. Yoshioka K. Takuwa N. (2012). Sphingosine-1-phosphate signaling in physiology and diseases. Biofactors 38, 329–337. 10.1002/biof.1030 22674845

[B92] Tayarani-Najaran Z. Tayarani-Najaran N. Eghbali S. (2021). A review of auraptene as an anticancer agent. Front. Pharmacol. 12, 698352. 10.3389/fphar.2021.698352 34239445 PMC8258114

[B93] Tu Y. Zhu S. Wang J. Burstein E. Jia D. (2019). Natural compounds in the chemoprevention of alcoholic liver disease. Phytother. Res. 33, 2192–2212. 10.1002/ptr.6410 31264302

[B94] Tuli H. S. Garg V. K. Bhushan S. Uttam V. Sharma U. Jain A. (2023). Natural flavonoids exhibit potent anticancer activity by targeting microRNAs in cancer: a signature step hinting towards clinical perfection. Transl. Oncol. 27, 101596. 10.1016/j.tranon.2022.101596 36473401 PMC9727168

[B95] Van Mourik H. Li M. Baumgartner S. Theys J. Shiri-Sverdlov R. (2022). All roads lead to cathepsins: the role of cathepsins in non-alcoholic steatohepatitis-induced hepatocellular carcinoma. Biomedicines 10, 2351. 10.3390/biomedicines10102351 36289617 PMC9598942

[B96] Wang H. Chen X. Calvisi D. F. (2021). Hepatocellular carcinoma (HCC): the most promising therapeutic targets in the preclinical arena based on tumor biology characteristics. Expert Opin. Ther. Targets 25, 645–658. 10.1080/14728222.2021.1976142 34477018 PMC8511244

[B97] Wang Y. Ding R. Zhang Z. Zhong C. Wang J. Wang M. (2021). Curcumin-loaded liposomes with the hepatic and lysosomal dual-targeted effects for therapy of hepatocellular carcinoma. Int. J. Pharm. 602, 120628. 10.1016/j.ijpharm.2021.120628 33892061

[B98] Wang C. L. Gao M. Z. Gao D. M. Guo Y. H. Gao Z. Gao X. J. (2022). Tubeimoside-1: a review of its antitumor effects, pharmacokinetics, toxicity, and targeting preparations. Front. Pharmacol. 13, 941270. 10.3389/fphar.2022.941270 35910383 PMC9335946

[B99] Wang S. Li W. Liu W. Yu L. Peng F. Qin J. (2023). Total flavonoids extracted from Penthorum chinense Pursh mitigates CCl(4)-induced hepatic fibrosis in rats via inactivation of TLR4-MyD88-mediated NF-κB pathways and regulation of liver metabolism. Front. Pharmacol. 14, 1253013. 10.3389/fphar.2023.1253013 38074148 PMC10701287

[B100] Wang Y. Chen Y. Y. Gao G. B. Zheng Y. H. Yu N. N. Ouyang L. (2023). Polyphyllin D punctures hypertrophic lysosomes to reverse drug resistance of hepatocellular carcinoma by targeting acid sphingomyelinase. Mol. Ther. 31, 2169–2187. 10.1016/j.ymthe.2023.05.015 37211762 PMC10362416

[B101] Wang J. Zhang J. Guo Z. Hua H. Zhang H. Liu Y. (2024). Targeting HSP70 chaperones by rhein sensitizes liver cancer to artemisinin derivatives. Phytomedicine 122, 155156. 10.1016/j.phymed.2023.155156 37897861

[B102] Wang Z. Wang G. Zhao P. Sun P. (2024). Multi-omics profiling and experimental verification of lysosomes-related genes in hepatocellular carcinoma. J. Cell Mol. Med. 28, e70225. 10.1111/jcmm.70225 39695350 PMC11655306

[B103] Wang C. Hu H. Gao H. Zhu Z. Zhao H. (2025). Astragali radix - curcumae rhizoma herb pair enhances Sorafenib's efficacy by inducing ferroptosis and activates Th1 cell immune response synergistically against hepatocellular carcinoma. Phytomedicine 148, 157326. 10.1016/j.phymed.2025.157326 41016296

[B104] Wang X. An F. Wang B. Zhang T. Fang Y. Yan L. (2025). From “lysosomal addiction” to targeted therapies: exploiting novel windows in colorectal cancer. Eur. J. Med. Res. 30, 957. 10.1186/s40001-025-03145-7 41074210 PMC12512390

[B105] Wei Z. Yan Y. Zhao L. Li C. Qi J. Chen D. (2025). Mitochondrial respiratory chain regulates HBV clearance through dual modulation of lysosomal acidification. Emerg. Microbes Infect. 14, 2563079. 10.1080/22221751.2025.2563079 41055213 PMC12507116

[B106] Wróbel M. Cendrowski J. Szymańska E. Grębowicz-Maciukiewicz M. Budick-Harmelin N. Macias M. (2022). ESCRT-I fuels lysosomal degradation to restrict TFEB/TFE3 signaling via the Rag-mTORC1 pathway. Life Sci. Alliance 5, e202101239. 10.26508/lsa.202101239 35354596 PMC8967991

[B107] Wu L. Weng Z. Yang X. Huang Y. Lin Y. Li S. (2025). ARL8B regulates lysosomal function and predicts poor prognosis in hepatocellular carcinoma. Sci. Rep. 15, 12278. 10.1038/s41598-025-97616-w 40210693 PMC11985964

[B108] Wu S. Anand N. Guo Z. Li M. Santiago Figueroa M. Jung L. (2025). Bridging immune evasion and vascular dynamics for novel therapeutic frontiers in hepatocellular carcinoma. Cancers (Basel) 17, 1860. 10.3390/cancers17111860 40507341 PMC12153674

[B109] Wu Y. P. Yang X. Y. Tian Y. X. Feng J. Yeo Y. H. Ji F. P. (2025). Dose-dependent relationship between alcohol consumption and the risks of hepatitis B virus-associated cirrhosis and hepatocellular carcinoma: a meta-analysis and systematic review. J. Clin. Transl. Hepatol. 13, 179–188. 10.14218/JCTH.2024.00379 40078198 PMC11894389

[B110] Xiao S. Peng K. Li C. Long Y. Yu Q. (2023). The role of sphingosine-1-phosphate in autophagy and related disorders. Cell Death Discov. 9, 380. 10.1038/s41420-023-01681-x 37852968 PMC10584985

[B111] Xiao F. Li H.-L. Yang B. Che H. Xu F. Li G. (2024). Disulfidptosis: a new type of cell death. Apoptosis 29, 1309–1329. 10.1007/s10495-024-01989-8 38886311 PMC11416406

[B112] Xie Q. Chen Y. Tan H. Liu B. Zheng L. L. Mu Y. (2021). Targeting autophagy with natural compounds in cancer: a renewed perspective from molecular mechanisms to targeted therapy. Front. Pharmacol. 12, 748149. 10.3389/fphar.2021.748149 34512368 PMC8427500

[B113] Xue L. Liu P. (2021). Daurisoline inhibits hepatocellular carcinoma progression by restraining autophagy and promoting cispaltin-induced cell death. Biochem. Biophys. Res. Commun. 534, 1083–1090. 10.1016/j.bbrc.2020.09.068 33213840

[B114] Yagi K. Shimada S. Akiyama Y. Hatano M. Asano D. Ishikawa Y. (2023). Loss of SFXN1 mitigates lipotoxicity and predicts poor outcome in non-viral hepatocellular carcinoma. Sci. Rep. 13, 9449. 10.1038/s41598-023-36660-w 37296228 PMC10256799

[B115] Yamashima T. (2025). Lysosomal membrane-permeabilization (LMP) and -rupture (LMR) are distinct for cell death. Front. Cell Death 4, 1669955. 10.3389/fceld.2025.1669955

[B116] Yamashita G. Takano N. Kazama H. Tsukahara K. Miyazawa K. (2022). p53 regulates lysosomal membrane permeabilization as well as cytoprotective autophagy in response to DNA-damaging drugs. Cell Death Discov. 8, 502. 10.1038/s41420-022-01293-x 36581628 PMC9800408

[B117] Yang M. Liu E. Tang L. Lei Y. Sun X. Hu J. (2018). Emerging roles and regulation of MiT/TFE transcriptional factors. Cell Commun. Signal 16, 31. 10.1186/s12964-018-0242-1 29903018 PMC6003119

[B118] Yang H. Wang J. Liu Z. G. (2022). Multi-faceted role of pyroptosis mediated by inflammasome in liver fibrosis. J. Cell Mol. Med. 26, 2757–2765. 10.1111/jcmm.17277 35415891 PMC9097829

[B119] Yang X. Wen Y. Liu S. Duan L. Liu T. Tong Z. (2022). LCDR regulates the integrity of lysosomal membrane by hnRNP K-stabilized LAPTM5 transcript and promotes cell survival. Proc. Natl. Acad. Sci. U. S. A. 119, e2110428119. 10.1073/pnas.2110428119 35091468 PMC8812561

[B120] Yang Y. Chen Y. Wu J. H. Ren Y. Liu B. Zhang Y. (2023). Targeting regulated cell death with plant natural compounds for cancer therapy: a revisited review of apoptosis, autophagy-dependent cell death, and necroptosis. Phytother. Res. 37, 1488–1525. 10.1002/ptr.7738 36717200

[B121] Yu H. Hao Z. Liu X. Wei Z. Tan R. Liu X. (2023). Autophagy blockage and lysosomal dysfunction are involved in diallyl sulfide-induced inhibition of malignant growth in hepatocellular carcinoma cells. Environ. Toxicol. 38, 2100–2110. 10.1002/tox.23834 37209385

[B122] Yu Q. Khanjyan M. Fidelman N. Pillai A. (2023). Contemporary applications of Y90 for the treatment of hepatocellular carcinoma. Hepatol. Commun. 7 (10), e0288. 10.1097/hc9.0000000000000288 37782464 PMC10545406

[B123] Yu D. Park J. Kim T. Choi C. Yuk S. A. Kim H. (2026). Nanotechnology-enabled delivery of phytochemicals: from formulation strategies to therapeutic translation. J. Phytomedicine 1, 4. 10.3390/jphytomed1010004

[B124] Yuan W. Jian F. Rong Y. (2022). Bifendate inhibits autophagy at multiple steps and attenuates oleic acid-induced lipid accumulation. Biochem. Biophys. Res. Commun. 631, 115–123. 10.1016/j.bbrc.2022.09.067 36183552

[B125] Yuan H. Liu J. Xu R. Yang K. Qu R. Liu S. (2025). The spatiotemporal heterogeneity of reactive oxygen species in the malignant transformation of viral hepatitis to hepatocellular carcinoma: a new insight. Cell Mol. Biol. Lett. 30, 70. 10.1186/s11658-025-00745-3 40517270 PMC12167593

[B126] Zarei M. H. Farzan M. Soleiman Dehkordi E. Lorigooini Z. Moradi M. T. (2023). The effect of infusion time on Echium amoenum extract -induced hepatotoxicity *in vitro* . Toxicon 229, 107133. 10.1016/j.toxicon.2023.107133 37127122

[B127] Zeng T. Zhou Y. Yu Y. Wang J. W. Wu Y. Wang X. (2023). rmMANF prevents sepsis-associated lung injury via inhibiting endoplasmic reticulum stress-induced ferroptosis in mice. Int. Immunopharmacol. 114, 109608. 10.1016/j.intimp.2022.109608 36700778

[B128] Zhang Y. Yang N. D. Zhou F. Shen T. Duan T. Zhou J. (2012). (-)-Epigallocatechin-3-gallate induces non-apoptotic cell death in human cancer cells via ROS-mediated lysosomal membrane permeabilization. PLoS One 7, e46749. 10.1371/journal.pone.0046749 23056433 PMC3466311

[B129] Zhang Y. S. Ma Y. L. Thakur K. Hussain S. S. Wang J. Zhang Q. (2018). Molecular mechanism and inhibitory targets of dioscin in HepG2 cells. Food Chem. Toxicol. 120, 143–154. 10.1016/j.fct.2018.07.016 29990575

[B130] Zhang H. Xu H. Tang Q. Bi F. (2021). The selective serotonin reuptake inhibitors enhance the cytotoxicity of sorafenib in hepatocellular carcinoma cells. Anticancer Drugs 32, 793–801. 10.1097/cad.0000000000001067 33675613

[B131] Zhang Y. P. Yang X. Q. Yu D. K. Xiao H. Y. Du J. R. (2021). Nrf2 signalling pathway and autophagy impact on the preventive effect of green tea extract against alcohol-induced liver injury. J. Pharm. Pharmacol. 73, 986–995. 10.1093/jpp/rgab027 33877365

[B132] Zhang J. Wu Y. Li Y. Li S. Liu J. Yang X. (2024). Natural products and derivatives for breast cancer treatment: from drug discovery to molecular mechanism. Phytomedicine 129, 155600. 10.1016/j.phymed.2024.155600 38614043

[B133] Zhang X. Chen Y. Li X. Xu H. Yang J. Wang C. (2024). Carrier-free self-assembled nanomedicine based on celastrol and galactose for targeting therapy of hepatocellular carcinoma via inducing ferroptosis. Eur. J. Med. Chem. 267, 116183. 10.1016/j.ejmech.2024.116183 38354520

[B134] Zhang N. Tian X. Liu F. Jin X. Zhang J. Hao L. (2025). Reversal of sorafenib resistance in hepatocellular carcinoma by curcumol: insights from network pharmacology, molecular docking, and experimental validation. Front. Pharmacol. 16, 1514997. 10.3389/fphar.2025.1514997 40242448 PMC12000033

[B135] Zhang R. Vooijs M. A. Keulers T. G. (2025). Key mechanisms in lysosome stability, degradation and repair. Mol. Cell Biol. 45, 212–224. 10.1080/10985549.2025.2494762 40340648 PMC12352500

[B136] Zheng Y. Ma Y. Xiong Q. Zhu K. Weng N. Zhu Q. (2024). The role of artificial intelligence in the development of anticancer therapeutics from natural polyphenols: current advances and future prospects. Pharmacol. Res. 208, 107381. 10.1016/j.phrs.2024.107381 39218422

[B137] Zhitomirsky B. Yunaev A. Kreiserman R. Kaplan A. Stark M. Assaraf Y. G. (2018). Lysosomotropic drugs activate TFEB via lysosomal membrane fluidization and consequent inhibition of mTORC1 activity. Cell Death Dis. 9, 1191. 10.1038/s41419-018-1227-0 30546014 PMC6294013

[B138] Zhou J. L. Huang X. Y. Qiu H. C. Gan R. Z. Zhou H. Zhu H. Q. (2020). SSPH I, a novel anti-cancer saponin, inhibits autophagy and induces apoptosis via ROS accumulation and ERK1/2 signaling pathway in hepatocellular carcinoma cells. Onco Targets Ther. 13, 5979–5991. 10.2147/OTT.S253234 32606806 PMC7320904

[B139] Zhou L. Li Y. Zheng D. Zheng Y. Cui Y. Qin L. (2024). Bispecific CAR-T cells targeting FAP and GPC3 have the potential to treat hepatocellular carcinoma. Mol. Ther. Oncol. 32, 200817. 10.1016/j.omton.2024.200817 38882528 PMC11179089

[B140] Zhu L. Hu J. Wu X. Zhang J. Xu X. Huang X. (2025). Programmed enhancement of endogenous iron-mediated lysosomal membrane permeabilization for tumor ferroptosis/pyroptosis dual-induction. Nat. Commun. 16, 3017. 10.1038/s41467-025-58124-7 40148335 PMC11950380

